# Chitosan-Based Polymeric Lipid Nanocapsules Loaded with *Cannabis sativa* L. Extract: Influence of Lipid Core Composition on Physicochemical Stability and Preliminary Anti-Chikungunya Virus Activity

**DOI:** 10.3390/polym18212701

**Published:** 2026-10-31

**Authors:** Luka Romano, Brenda Salome Konigheim, Juan Javier Aguilar, Esteban Martin Schenfeld, Mariana Quiroga, Marcelo Helguera, Gladys Ester Granero, Renée Onnainty

**Affiliations:** 1 Unidad de Investigaciones y Desarrollo en Tecnología Farmacéutica (UNITEFA) CONICET, Departamento de Ciencias Farmacéuticas, Facultad de Ciencias Químicas, Universidad Nacional de Córdoba, Córdoba X5000, Argentina; 2 Instituto de Virología “Dr. José María Vanella” (InViV), Facultad de Ciencias Médicas, Universidad Nacional de Córdoba, Córdoba X5000, Argentina; 3 Consejo Nacional de Investigaciones Científicas y Técnicas (CONICET), Buenos Aires C1425, Argentina; 4 Unidad de Estudios Agropecuarios (UDEA) (INTA-CONICET), Instituto de Fisiología y Recursos Genéticos Vegetales (IFRGV), Córdoba X5020, Argentina

**Keywords:** *Cannabis sativa* extract, chitosan nanocapsules, encapsulation efficiency, lipid core composition, Box–Behnken design, ATR-FTIR, chikungunya virus

## Abstract

Cannabidiol (CBD), the main non-intoxicating phytocannabinoid of *Cannabis sativa*, is highly lipophilic and poorly water-soluble. We developed chitosan-coated lipid nanocapsules (NCs) loaded with a CBD-rich *Cannabis sativa* ethanolic extract (CEE), using medium-chain triglyceride (MCT) oil or olive oil as the lipid core and optimizing them with a Box–Behnken design; oil and Tween^®^ 80 volumes mainly determined hydrodynamic diameter and polydispersity. The optimized MCT oil and olive oil NCs had hydrodynamic diameters of 206.9 and 231.2 nm, PDI values of 0.130 and 0.196, zeta potentials of about +31 and +33 mV, and CBD encapsulation efficiencies above 85%. ATR-FTIR suggested different lipid organization and CBD environments in each core, while confocal microscopy located the CBD-reactive signal in the oil phase without resolving the core from the interface. MCT oil NCs retained more CBD at room temperature, but differences under other conditions were small, and the extract dissolved in the same oils behaved similarly, indicating a major contribution of the oil vehicle. Both NCs retained CBD between pH 5.5 and 7.4 and could be redispersed after freeze-drying. In a preliminary chikungunya virus assay, CEE-loaded NCs inhibited the cytopathic effect by 34–45% at non-cytotoxic concentrations, but free CEE in oil was at least as active.

## 1. Introduction

Extracts of *Cannabis sativa* L. contain cannabidiol (CBD), as well as other phytocannabinoids, terpenes, and phenolic constituents [1]. CBD has attracted considerable interest due to its reported anti-inflammatory, antioxidant, and immunomodulatory properties [2]. However, its high lipophilicity, poor aqueous solubility, and extensive first-pass metabolism result in low and variable oral bioavailability [3], and the composition of complex extracts varies with genetic, environmental, and extraction-related factors [1]. To limit this variability, we used a *Cannabis sativa* ethanolic extract (CEE) from a defined, CBD-rich genetic line grown under controlled conditions, so that CBD could serve as a reliable marker compound. Nanocarriers have been explored to overcome these limitations [4,5], but most studies have focused on isolated CBD or Δ^9^-tetrahydrocannabinol rather than full-spectrum extracts, for which standardized quality-control data remain scarce [6].

Lipid–polymer hybrid nanocapsules (NCs) combine a liquid hydrophobic reservoir with a polymer-mediated interface. Unlike liposomes or solid lipid nanoparticles, the liquid core can dissolve a complex lipophilic mixture without a crystalline lipid matrix, and the polymer shell stabilizes the interface [7,8]. In solvent-displacement methods, mixing the organic and aqueous phases promotes lipid dispersion via solvent diffusion, after which the polymer associates with or deposits on the newly formed interface to stabilize the shell [7,9]. This association is expected to rely on non-covalent interactions (electrostatic attraction, hydrogen bonding, hydrophobic contacts). The balance of interactions among lipid, surfactant, polymer, and payload therefore determines hydrodynamic diameter, polydispersity, surface charge, and loading [7,8].

Several other approaches could be used to obtain nanocarriers for lipophilic compounds. Emulsification–solvent evaporation, emulsification–diffusion, and double-emulsion methods generally involve an additional high-energy emulsification step and organic solvents that must subsequently be removed, whereas high-pressure homogenization, typical of solid lipid nanoparticles, relies on specialized equipment and, in its hot version, on elevated temperatures that may be unsuitable for thermolabile compounds [10,11]. Ionic gelation of chitosan with tripolyphosphate is a mild, purely aqueous method, but it has mainly been applied to hydrophilic payloads such as proteins and does not provide a liquid lipid core able to dissolve a lipophilic, chemically complex mixture [12]. By contrast, solvent displacement is a simple, one-step, low-energy procedure performed at room temperature, in which an acetone phase containing the oil, surfactant, and extract is injected into an aqueous chitosan solution and the oil core and polymeric shell form spontaneously [11,13]. These features make it the most suitable approach for a thermolabile, oily, full-spectrum extract, and it is the procedure our group previously established for chitosan-coated nanocapsules [14].

Chitosan (CS), a polysaccharide of glucosamine and N-acetylglucosamine units, is widely used to build such shells [15,16]. In acidic media, its primary amino groups are protonated, increasing aqueous solubility and conferring a positive surface charge [15,17]. However, CS is not uniform: its molecular weight (MW) and degree of deacetylation (DD) determine chain length, solution viscosity, solubility, and the density of protonatable amino groups [17]. Higher MW increases viscosity and may favor more extended or entangled polymer layers, increasing hydrodynamic diameter and aggregation risk, whereas higher DD increases the fraction of protonatable amino groups [17]. In chitosan-tripolyphosphate particles, for instance, the degree of deacetylation and ionic strength influence particle size [18], and surface charge depends on pH, ionic strength, and polymer coverage [19]. As these relationships are system-dependent, MW and DD should be reported as key material attributes rather than assumed to universally govern shell properties.

The present study used medium-MW CS (MW ~ 190,000–310,000 Da; Sigma-Aldrich; DD 82–93% by ^1^H NMR and ATR-FTIR; supplier: 75–85%), the grade previously used by our group to build polyelectrolyte layers [20], selected as a compromise between processability in dilute aqueous acetic acid and availability of cationic amino groups to interact with the lipid/surfactant interface. Because different CS grades were not compared, MW and DD are treated as design considerations rather than experimentally isolated variables.

The lipid core is a second determinant of NC performance. Medium-chain triglyceride (MCT) oil and olive oil differ in fatty-acid chain length and unsaturation, which affect the fluidity and oxidative susceptibility of the oil phase and the microenvironment of a lipophilic payload [21,22]. Lipid composition and polymer–lipid interactions also influence particle formation, loading, and stability [7,9,21], yet direct comparisons of MCT and olive oil in CS-coated NCs containing a chemically complex *Cannabis sativa* extract are lacking.

Cannabinoids have attracted interest in their immunomodulatory and antiviral properties, and CBD has shown activity against enveloped and non-enveloped viruses [23]. Chikungunya virus (CHIKV) is an emerging alphavirus with no specific treatment. In vitro, CBD modulated natural killer and monocytic cell subsets during CHIKV exposure [24] but did not significantly reduce CHIKV replication in human macrophages, whereas the synthetic cannabinoid WIN 55,212-2 did [25]. Immunomodulation should therefore not be equated with direct antiviral activity, and the effect of CBD-containing nanocapsules on CHIKV remains to be examined; the antiviral assay was therefore a preliminary screening.

This study aimed to develop and characterize CS-coated polymeric lipid NCs loaded with CEE, using MCT or olive oil as the lipid core. We hypothesized that the lipid core, together with CS and Tween^®^ 80 content, governs the colloidal properties of the NCs and modulates CBD retention and chemical stability. The formulations were optimized using a Box–Behnken design [26,27] and compared in terms of colloidal properties, CBD encapsulation, and stability under storage and stress conditions. ATR-FTIR and confocal microscopy with the CBD-reactive dye Fast Blue were used to probe where CBD is located within the NCs; as these techniques provide only indirect or low-resolution evidence, differences in CBD localization between lipid matrices are considered a mechanistic hypothesis rather than a direct structural measurement. Finally, antiviral activity against CHIKV was screened at non-cytotoxic concentrations.

## 2. Materials and Methods

### 2.1. Materials

Chitosan (CS) of medium molecular weight (MW ~ 190,000–310,000 Da) was purchased from Sigma-Aldrich (Buenos Aires, Argentina). According to the supplier, the deacetylation degree (DD) of CS was 75–85%. Here, the DD was also determined by ^1^H NMR and ATR-FTIR techniques, and it was found to be 93% and 82%, determined by NMR and ATR-FTIR, respectively [28,29], previously determined and reported for the same CS grade [20]. Tween^®^ 80 was purchased from Sigma-Aldrich (Buenos Aires, Argentina); acetic acid, methanol, and ethanol (Sintorgan, Proveeduría Científica, Córdoba, Argentina) were used without further purification. Analytical-grade solvents were also used. MCT (medium-chain triglyceride) oil and olive oil (food grade, Córdoba, Argentina) were used as lipid cores to prepare the NCs. Ultrapure water was obtained using a Milli-Q purification system. The ethanolic extract of *Cannabis sativa* L. (CEE) was obtained from the experimental line “Cogollos INTA CBD,” a defined CBD-rich genetic line, grown under controlled conditions in a UDEA-IFRGV-CIAP/INTA-CONICET facility under an INTA-Asoc. Civil Cogollos Córdoba agreement and stored in the dark until use. The cannabidiol (CBD) content was 48% in CEE, quantified by HPLC. All experiments used extract from a single batch, so differences between formulations could be attributed to the lipid core and formulation variables rather than variability in extract composition.

### 2.2. Preparation of Cannabis Ethanolic Extract (CEE)

Freshly harvested inflorescences (1000 g) were uniformly distributed onto a heat-resistant tray to ensure homogeneous thermal transfer. The vegetal material was decarboxylated in an oven at 110 °C for 30 min to ensure high conversion of acidic phytocannabinoids into their active neutral forms [30,31]. Following treatment, the material was allowed to cool to room temperature.

Subsequently, a solid-to-solvent ratio of 1:10 (*w*/*v*) was established in an appropriate glass vessel. Before solid–liquid contact, both the vegetal material and ethanol were pre-chilled to sub-zero temperatures using liquid nitrogen to minimize solubility and co-extraction of waxes and chlorophylls. Extraction was carried out by adding cold ethanol under gentle, constant agitation for 10 min, keeping the temperature as low as possible throughout the process.

After extraction, the mixture was immediately filtered through filter paper. The resulting extract was stored in a sealed container at −20 °C for 24 h. The following day, without allowing the extract to reach room temperature, a second filtration was performed using fine-grade filter paper to remove the precipitated material. The clarified extract was then transferred to a rotary evaporator and concentrated under reduced pressure at 40 °C until ethanol was completely removed, yielding a phytocannabinoid-rich concentrate.

The HPLC analysis of the CEE confirmed CBD as its major phytocannabinoid in the sample, although the relative phytocannabinoid composition of plant-derived extracts may vary between batches; CBD consistently remained the predominant compound.

### 2.3. Selection of Lipid Core

Initially, the compatibility of various vegetable oils with the CEE was evaluated, along with their suitability for NC formation. Solubility tests and preliminary HPLC analyses were conducted on sunflower oil, corn oil, castor oil, olive oil, and MCT oil.

Olive oil and MCT oil were selected as lipid cores because they fully solubilized the extract and were compatible with the formulation components, showing no chromatographic interference or phase separation.

### 2.4. Preparation of Polymeric Nanocapsules

Polymeric NCs were prepared by the solvent displacement technique, following our previous protocol [14]. Briefly, the organic phase comprised 2 mL of the selected oil (MCT or olive oil), Tween^®^ 80, and CEE, dissolved in acetone via magnetic stirring. The aqueous phase (5 mL) was prepared by dissolving CS in an aqueous acetic acid solution (0.1%p/v) under continuous stirring until complete solubilization. The organic phase was then rapidly injected into the aqueous phase at room temperature and under rapid stirring to promote the spontaneous formation of nanocapsules (NCs) through solvent displacement and interfacial polymer deposition. Once the nanoparticles had formed, the acetone was evaporated by leaving the solutions under a fume hood with continuous stirring overnight. The formulations were then adjusted to the desired final volume with ultrapure water. The NCs were then separated from the reaction media by centrifugation at 21,130 RCF for 15 min at 4 °C. Empty NCs were prepared using the same procedure but without adding CEE.

### 2.5. Experimental Design and Formulation Optimization

A Box–Behnken factorial design was used to optimize the NC formulation parameters.

The independent variables were CS concentration (0.5, 1.25, and 2.0 mg/mL), oil volume (50, 100, and 150 µL), and Tween^®^ 80 volume (25, 50, and 75 µL). The design comprised 15 runs, including three center points. The response variables selected were particle hydrodynamic diameter, polydispersity index (PDI), and zeta potential. The experimental design and statistical analysis were performed using response surface methodology (RSM) with Minitab^®^ (Minitab 18, LLC, State College, PA, USA). A full quadratic model (three main effects, three quadratic terms, and three two-factor interactions) was fitted to each response, and effects were considered significant at α = 0.05 (t = 2.57, 5 error degrees of freedom). Pareto charts and three-dimensional response surface plots were generated to evaluate the effects of the formulation variables, both individually and in combination, on NC properties. The optimized formulations were selected based on a hydrodynamic diameter close to 200 nm, a PDI below 0.25, and a zeta potential above +30 mV.

### 2.6. Dynamic Light Scattering (DLS) and Zeta Potential Analysis

The hydrodynamic diameter, polydispersity index (PDI), and zeta potential of the NCs were determined using a Zetasizer Nano instrument (Malvern Instruments, Malvern, UK) via dynamic light scattering (DLS) and electrophoretic mobility measurements. Samples were diluted with ultrapure water prior to analysis to avoid the effects of multiple scattering. Measurements were performed in triplicate at 25 °C. Particle hydrodynamic diameter distributions were analyzed by intensity, volume, and number.

### 2.7. Scanning Electron Microscopy (SEM)

The morphology and surface characteristics of the NCs were evaluated using scanning electron microscopy (SEM) on a Carl Zeiss Sigma (Zeiss, Oberkochen, Germany) analytical scanning electron microscope at an accelerating voltage of 5 kV (LAMARX laboratory, FAMAF, UNC). The samples were deposited onto metallic supports and dried under controlled conditions. Before observation, the dried materials were coated with a thin conductive metallic layer. SEM images were obtained in a high-vacuum environment at various magnifications to evaluate particle morphology, surface homogeneity, and aggregation behavior.

### 2.8. ATR-FTIR Spectroscopy and Spectral Deconvolution

ATR-FTIR (FT-IR, Agilent Cary 630, ATR diamond crystal) analyses were performed on pure CEE, empty NCs, CEE-loaded MCT oil NCs, and CEE-loaded olive oil NCs, and the respective lyophilized samples.

Spectra were recorded using an ATR-FTIR spectrometer over the range 4000–600 cm^−1^ with a resolution of 4 cm^−1^, and 64 scans were accumulated, with a 30 s measurement time. Spectral deconvolution was then performed to improve resolution of overlapping bands, particularly in the fingerprint region (1800–600 cm^−1^). Peak assignment was based on the characteristic vibrational frequencies of cannabinoids, triglycerides, CS, and surfactant molecules reported in the literature. A comparative analysis of the deconvoluted spectra was then performed to investigate the intermolecular interactions between the encapsulated cannabinoids and the NC matrix.

### 2.9. Confocal Laser Scanning Microscopy (CLSM) with Fast Blue

The spatial distribution of CBD within the NCs was examined by confocal laser scanning microscopy (CLSM) using Fast Blue BB salt (Fluka, Chemika), a diazonium salt that couples with the phenolic ring of cannabinoids to give coloured azo products [32], as a CBD-reactive probe. Because it reacts with phenolic hydroxyl groups, Fast Blue is CBD-reactive but not strictly CBD-specific [32]. Nanocapsule suspensions (10 µL) were deposited onto glass coverslips and incubated with a 0.1% (*w*/*v*) Fast Blue BB solution in acid methanol for 5 min at room temperature. After incubation, coverslips were washed twice with water and mounted onto glass slides. Empty nanocapsules, chitosan solution, and olive and MCT oil without extract were processed identically and used as negative controls, as was a Fast Blue BB solution without nanocapsules (dye-alone control). An aliquot of each sample was placed between a glass slide and a coverslip and imaged immediately.

Images were acquired on a Nikon Eclipse Ti CS1 confocal laser scanning microscope (EZ-C1 Silver v3.91) equipped with a Plan Apo VC 60×/1.40 NA oil-immersion objective (Nikon Instruments, Tokyo, Japan) (UDEA-IFRGV-CIAP/INTA-CONICET). The 488 nm line of an Ar laser (80.8% power) was used for excitation. Fluorescence emission was collected simultaneously in two channels: Ch2 (515/30 nm bandpass filter) and Ch3 (605/75 nm bandpass filter), corresponding to the dual emission of the azo complex Fast Blue–CBD. A transmitted-light channel (Trans) was acquired in parallel to provide morphological context. The dichroic mirror configuration was DM480/DM545. Images were collected at 1024 × 1024 pixels (pixel size 0.02–0.21 µm; field of view 18–212 µm depending on the image, Table S6), with a pinhole of 153.8 µm and a pixel dwell time of 6 µs.

CBD-loaded NCs, empty NCs, and controls were imaged with the same laser power and offset and with detector gains of 6.5–6.8 (Table S6), and are displayed with identical intensity scaling.

Image analysis was performed in Python 3.9 (NumPy, SciPy, scikit-image). The background of each channel was estimated as its 5th intensity percentile and subtracted; signal intensity was expressed as the 99th percentile of the background-subtracted image. Given the small number of images per condition (Table S6), the analysis is descriptive.

### 2.10. High-Performance Liquid Chromatography (HPLC)

High-performance liquid chromatography (HPLC) was used to quantify CBD from CEE on a JASCO LC-2000Plus HPLC system (Jasco, Tokyo, Japan). The system was equipped with a PU-2089S Plus quaternary gradient pump and a UV-2077S Plus 4λ Intelligent UV/Vis Detector operating at 228 nm. Chromatographic separation was performed on a PerkinElmer Epic C18 column (150 × 4.6 mm, 3 µm, 120 Å) (PerkinElmer, Waltham, MA, USA) in reverse-phase mode. The mobile phase consisted of acetonitrile (J.T.Baker, OneLab, Buenos Aires, Argentina) and acidified water (80:20 *v*/*v*) containing 1% (*v*/*v*) formic acid (Anedra, Proveeduria Científica, Córdoba, Argentina). Before using it, the mobile phase was filtered and degassed. Analyses were performed at a flow rate of 1 mL/min, with the column temperature maintained at 37 °C for 15 min. The injection volume was 10 μL.

### 2.11. Determination of Encapsulation Efficiency

A direct method was developed to determine the encapsulation efficiency (EE%) of the CEE. Preliminary HPLC analysis of the CEE confirmed CBD as its major phytocannabinoid; based on this, CBD content was used as a marker for total CEE encapsulation and was quantified using a previously developed HPLC method. The total CBD added to each formulation (CBD*_total_*) was calculated from the mass of CEE used and its CBD content, determined by HPLC. After preparation, the non-encapsulated material was removed by centrifugation (Section 2.4), so the CBD quantified afterward came exclusively from the NCs. Then, 500 μL of the NC suspension was mixed with 1 mL of acetone to disrupt the nanocapsule structure and release the encapsulated CBD. The resulting mixtures were filtered through 0.2 μm syringe filters directly into HPLC vials before analysis. Encapsulation efficiency was calculated according to Equation (1):

(1) $$\mathrm{EE}\;\left(\%\right)=\;\left[\frac{{\mathrm{CBD}}_{\mathrm{load}}}{{\mathrm{CBD}}_{\mathrm{total}}}\right]\times \;100$$

where CBD*_total_* corresponds to the total amount of cannabidiol added to the formulation, and CBD*_load_* corresponds to the CBD recovered from the nanocapsules after removal of the non-encapsulated fraction. All measurements were performed in triplicate.

CBD in the CEE solubilized in each oil was quantified in the same way after dilution in acetone. CBD quantification was performed using calibration curves prepared from analytical CBD standards under the same chromatographic conditions. All analyses were carried out in triplicate.

### 2.12. Freeze-Drying and Redispersion Studies

The NCs were converted into solid-state formulations by freeze-drying, with sucrose serving as the cryoprotective agent [33]. Briefly, 1 mL of the NC suspension was mixed with 1 mL of a 10% (*w*/*v*) sucrose solution in 5 mL transparent glass vials. The vials were covered with filter paper to facilitate sublimation during lyophilization. The samples were rapidly frozen in liquid nitrogen at approximately −200 °C, then freeze-dried in the dark using a Labconco FreeZone 6 lyophilizer (Labconco, Kansas City, MO, USA). Lyophilization was performed at −40 °C for 48 h under pressure below 120 µbar to obtain uniform dry cakes. After freeze-drying, the NCs were resuspended in 5 mL ultrapure water and analyzed by DLS for hydrodynamic diameter, PDI, and zeta potential. These analyses evaluated how lyophilization affected the NCs’ physicochemical properties and assessed their redispersion capacity after rehydration.

### 2.13. Stability Studies of Non-Lyophilized Nanocapsules

Long-term and forced stability studies evaluated the NCs’ physicochemical stability and the encapsulated CBD’s chemical stability. For both studies, comparisons were made between CBD encapsulated in NCs and CBD solubilized in the oil phase used to form the NC core. Because CEE is a lipophilic, oily extract immiscible with water, a control of free CEE in an aqueous medium was not feasible, as phase separation prevents representative sampling and quantification. CEE solubilized in the corresponding oil, which mimics the lipid core of the NCs, was therefore used as the reference matrix. For the same reason, the stability of free CEE at different pH values could not be evaluated.

The stability study was designed as a comparative, laboratory-scale assessment inspired by the ICH Q1A(R2) [34] and Q1B guidelines [35], and not as a registration stability study. Long-term storage was evaluated at 4 °C (refrigerated, 5 ± 3 °C in ICH Q1A(R2)) and at room temperature (25 ± 2 °C) for 30 days, with sampling on days 0, 10, 20 and 30 (*n* = 3 independent preparations/samples per condition). Stress conditions included thermal stress at 50 °C and UV exposure, with sampling at 0, 30, 60, 120, and 240 min. Relative humidity was not controlled, since the samples were aqueous suspensions in sealed vials. (UV source: Yarlux, 15 W, 437 mm, UV-C wavelength 253.7 nm, irradiation at 10 cm).

#### 2.13.1. Long-Term Stability Studies

For the long-term stability experiments, 3 mL of the NC suspension and 3 mL of 5% (*w*/*v*) CEE solubilized in the corresponding oil phase were stored separately in sealed amber glass vials, then sealed with Parafilm. Samples were stored at room temperature and at 4 °C. Aliquots of 500 µL were collected on days 0, 10, 20, and 30. At each time point, 30 µL of the sample was analyzed by DLS for particle hydrodynamic diameter and PDI. The remaining 470 µL was mixed with 1 mL of acetone to disrupt the NC structure and release the encapsulated CBD for HPLC analysis.

#### 2.13.2. Forced Stability Studies

Forced stability studies were conducted on independent 500 µL samples placed in transparent glass vials and sealed with Parafilm. The samples were exposed to two stress conditions: thermal stress at 50 °C and ultraviolet (UV) light exposure. Aliquots were collected at 0, 30, 60, 120, and 240 min. At each time point, 30 µL of the suspension was used for DLS measurements to assess changes in particle hydrodynamic diameter and PDI. The remaining 470 µL was treated with 1 mL of acetone to disrupt the NCs, then analyzed by HPLC to quantify CBD content. All stability experiments were performed under controlled conditions and protected from external contamination.

### 2.14. In Vitro Stability Studies at Different pH Using Franz Diffusion Cells

The in vitro stability of the NCs under different pH conditions was evaluated using horizontal Franz diffusion cells. Semi-permeable cellulose dialysis membranes (Dialysis Tubing Cellulose Membrane, molecular weight cut-off 14 kDa, Sigma-Aldrich, Buenos Aires, Argentina) were placed between the donor and receptor compartments. The donor and receptor compartments were filled with phosphate-buffered saline (PBS) at pH 5.5, 6.8, or 7.4, depending on the experimental conditions. The system was kept under constant stirring at 37 °C in both compartments using a thermostatically controlled circulating water bath. Samples were collected from the receptor compartment at 0, 30, 60, 120, and 240 min using plastic syringes. After each sampling, the removed volume was replaced with fresh medium to maintain a constant volume and sink conditions throughout the experiment. The collected samples were subsequently analyzed by HPLC to evaluate the possible diffusion or release of CBD through the membrane under the different pH conditions.

### 2.15. Biological Activity

Unless otherwise stated, all concentrations in this section are expressed as cannabidiol (CBD) concentration, as quantified by HPLC, both for the CEE-loaded NCs and for the free CEE dissolved in oil. For the empty NCs, concentrations correspond to the same dilutions of the corresponding CEE-loaded formulation (CBD-equivalent).

#### 2.15.1. Cell Culture and Viruses

The host cells, African green monkey kidney cells (*Chlorocebus aethiops*, Vero cl76 obtained from Laboratory of Cell Culture-Instituto de Virología “Dr. J. M. Vanella”, Córdoba, Argentina), were propagated in Eagle’s minimal essential medium (MEM) (Gibco, Grand Island, NY, USA) supplemented with 10% (*v*/*v*) fetal bovine serum (FBS, Natocor, Carlos Paz, Argentina), L-glutamine (30 μg/mL, Sigma-Aldrich), and gentamicin (50 μg/mL, Sigma-Aldrich).

Infection studies were performed with the chikungunya virus (CHIKV) ARG strain 2016 (GenBank accession no. OQ852708.1) [36].

#### 2.15.2. In Vitro Cytotoxicity Assay

To determine formulation concentrations that did not affect host cell viability, a neutral red (NR; Gibco, USA) uptake assay was performed following a previously described procedure [37]. Briefly, confluent Vero cell monolayers (1 × 10^5^ cells/well; approximately 95% confluence) cultured in 96-well plates were exposed to eleven concentrations in serial dilutions (dilution factor ≈ 1.75), starting at a CBD concentration of 0.103 mg/mL for the CEE-loaded MCT oil NCs or 0.107 mg/mL for the CEE-loaded olive oil NCs. Empty MCT oil NCs and empty olive oil NCs were evaluated in parallel as vehicle controls (at the same dilutions as the corresponding CEE-loaded NCs), and CEE dissolved in MCT oil or in olive oil was tested as a free-extract reference, starting at a CBD concentration of 0.25 mg/mL (quantified by HPLC) with serial dilutions of ≈1.5-fold.

Three replicate wells were analyzed for each concentration, and three wells with cells exposed to culture medium alone were included as the cell control (CC). After 72 h of incubation at 37 °C and 5% CO_2_, the absorbance of the NR extracted from the cells was measured at 540 nm using a microplate reader (BioTek ELx800, Winooski, VT, USA). Cell viability was expressed as a percentage relative to the CC, which was considered 100% viability. The assay was performed in two independent experiments.

#### 2.15.3. Antiviral Activity by Inhibition of the Cytopathic Effect

Antiviral activity was assessed following the methodology described by Watanabe et al. [38], with minor modifications. Briefly, cell monolayers were established by seeding Vero cells in 96-well plates for 24 h. Subsequently, 100 µL/well of each formulation was added at CBD concentrations previously determined to be non-cytotoxic in the cytotoxicity assay (≥70% cell viability). Immediately thereafter, the cells were infected with 20 µL/well of CHIKV (Multiplicity of Infection, MOI 0.1). Uninfected, untreated cells served as the cell control (CC), whereas infected, untreated cells served as the viral control (VC) and uninfected, treated cells served as the formulation control (FC). Given that there is currently no approved specific antiviral treatment for CHIKV infection, no reference antiviral compound was included in this assay as a positive control. The cells were then incubated for 72 h at 37 °C and 5% CO_2_ in a humidified atmosphere. The assay was terminated when inverted light microscopy revealed 100% cytopathic effect (CPE) in all viral control wells. The extent of CPE was quantified using the NR uptake assay, following the same procedure described above for the cell viability assay. The percentage of CPE inhibition was calculated according to the following equation:

% CPE inhibition = (OD_sample_ − OD_vc_)/(OD_FC_ − OD_VC_) × 100 (2)

where: OD_sample_ represents the absorbance of cells infected with CHIKV and treated with the corresponding formulation, OD_VC_ represents the absorbance of the viral control, and OD_FC_ represents the absorbance of the formulation control.

Differences were analyzed by Dunnett’s test against the corresponding controls and by two-way ANOVA (sample × concentration) with Holm-adjusted pairwise comparisons (*p* < 0.05).

By adding the formulations immediately before viral inoculation, this experimental design allows an overall assessment of their potential antiviral activity from the initial stages of infection onward, without restricting the evaluation to a specific stage of the viral replication cycle. However, this approach provides an overall measure of antiviral activity and does not allow determination of the specific stage or mechanism of viral inhibition.

### 2.16. Statistical Analysis

All experiments were performed in triplicate, and results are presented as the mean ± standard deviation. Statistical analyses were performed using analysis of variance (ANOVA), and differences were considered statistically significant at *p* < 0.05. The optimization study was analyzed using response surface methodology and Pareto analysis.

## 3. Results and Discussion

### 3.1. Selection of Lipid Core and Solubilization of Cannabinoids

As a critical step in designing the NC lipid core, we evaluated the solubility of the CEE in different Córdoba vegetable oils (Figure 1). Of the oils tested, olive oil and MCT oil fully solubilized the extract and allowed for the appropriate chromatographic resolution of cannabinoids. In contrast, sunflower and corn oils caused interferences during HPLC analysis, and castor oil formed phases and was incompatible with the formulation system.

The lipid phase selection is fundamental to the performance of polymeric NCs, directly affecting drug loading, encapsulation efficiency, colloidal stability, and the chemical protection of lipophilic compounds. Previous studies have shown that lipid matrices containing medium-chain triglycerides are more effective at solubilizing hydrophobic molecules than long-chain triglyceride systems due to their lower viscosity and higher diffusional mobility [21].

In the present study, both olive oil and MCT oil were highly compatible with the CBD-rich extract. However, MCT oil showed improved physicochemical behavior during NC formation, likely due to its lower viscosity and greater ability to stabilize small lipid droplets during solvent displacement.

### 3.2. Optimization of Nanocapsule (NC) Formulation

A Box–Behnken experimental design was used to optimize formulation parameters affecting the physicochemical properties of NCs, prepared using either olive oil or MCT oil as the lipid core. The independent variables were oil volume, Tween^®^ 80 volume, and CS concentration, while hydrodynamic diameter, PDI, and zeta potential were selected as response variables. This statistical approach allowed us to evaluate the individual and interactive effects of the formulation components on the colloidal characteristics of both nanosystems simultaneously. Figure 2 and Figure 3 show the results for the olive oil formulations.

Pareto charts showed that, for olive oil formulations, oil volume had the largest effect on hydrodynamic diameter and PDI, followed by Tween^®^ 80 volume and, for hydrodynamic diameter, CS concentration (Figure 2A,B); none of the terms reached statistical significance for zeta potential (Figure 2C). A comparable trend was observed for the MCT oil formulations. Increasing the Tween^®^ 80 volume promoted the formation of smaller, more homogeneous nanoparticles (NPs) by stabilizing lipid droplets during solvent displacement. Tween^®^ 80 reduces the interfacial tension between the organic and aqueous phases, thereby limiting droplet coalescence during nanoparticle nucleation. Similar behavior has been widely reported for polymeric NCs prepared by interfacial deposition methods [39].

However, differences were observed between the two lipid systems. In the optimized formulations, MCT oil NCs showed a smaller hydrodynamic diameter and a narrower distribution than olive oil NCs (Section 3.3). This may be due to MCTs’ lower viscosity and shorter hydrocarbon chains, which facilitate more efficient emulsification and the formation of smaller droplets during nanoparticle assembly. In contrast, the higher proportion of long-chain unsaturated triglycerides in olive oil may increase internal-phase viscosity and promote larger droplets before CS deposition. Similar effects of lipid composition on nanoparticle formation have been described in lipid–polymer hybrid nanocarriers [4].

CS concentration significantly affected hydrodynamic diameter in both systems, as a main effect and through its interaction with Tween^®^ 80 volume. Higher polymer concentration increased particle size by raising aqueous-phase viscosity and forming thicker polymeric shells around the lipid core. This effect may be more pronounced in olive oil NCs, possibly because olive oil’s higher viscosity, combined with the elevated polymer concentration, further limits diffusion during solvent displacement. Similar observations have been reported for CS-coated nanostructures, where increased polymer concentration favors particle growth and broader size distributions [18].

The Pareto analysis indicated that achieving stable nanosystems with low dispersity requires balancing the oil and Tween^®^ 80 volumes (significant oil × Tween^®^ 80 interaction), while CS concentration affected PDI mainly through its quadratic term (Figure 2B). At low Tween^®^ 80 volumes, particularly in formulations with higher olive oil volumes, partial aggregation and broader particle distributions were observed. Conversely, MCT oil systems tolerated formulation changes better, maintaining relatively low PDI values across a wider experimental range.

Response surface plots (Figure 3) provided additional insight into how formulation variables jointly influenced NC properties. The three-dimensional response surfaces for hydrodynamic diameter (Figure 3A) showed that increasing Tween^®^ 80 concentration progressively reduced particle diameter in both systems, although the reduction was more pronounced for MCT oil NCs. This behavior suggests that MCT exhibits more efficient interfacial stabilization under high-surfactant conditions.

Oil volume had the largest effect on hydrodynamic diameter (Figure 2A; the surfaces in Figure 3 are shown at the central oil volume of 100 µL), probably because larger initial emulsion droplets formed before polymer deposition. This effect may be more pronounced in olive oil formulations, given olive oil’s greater viscosity and molecular complexity than MCT oil.

Response surface analysis of PDI (Figure 3B; oil volume fixed at 100 µL) showed that low dispersity values were achieved at high Tween^®^ 80 volumes, and that PDI varied with CS concentration in a curved fashion, with the highest values at intermediate concentrations (significant quadratic term, Figure 2B). Under these conditions, rapid lipid-droplet stabilization prevents coalescence and favors the formation of homogeneous NC populations. Once again, MCT oil systems displayed more regular response surfaces and broader regions of low PDI values, indicating improved formulation robustness compared with olive oil NCs.

Interestingly, the response surfaces also revealed curvature effects, confirming nonlinear relationships between the formulation variables and NCs properties. This finding supports the applicability of response surface methodology (RSM) for optimizing polymeric NCs, as it enables simultaneous evaluation of interactions and quadratic effects [27].

As shown in Figure 3C, the zeta potential surface showed a trend toward higher positive surface charges with increasing CS concentration (from ~37–43 mV at 0.5 mg/mL to ~52 mV at 2.0 mg/mL), consistent with more protonated amino groups on the NC surface; this effect, however, did not reach statistical significance (Figure 2C). Most optimized systems showed positive zeta potential values above +30 mV, indicating strong electrostatic stabilization and reduced aggregation. However, olive oil formulations occasionally showed slightly lower zeta potential values at elevated oil concentrations, potentially because larger lipid droplets reduced the effective coverage of the CS surface.

Overall, the combined Pareto analysis and response surface methodology showed that surfactant concentration primarily governs hydrodynamic diameter and homogeneity, while CS concentration predominantly controls surface charge and structural stabilization. Importantly, a comparative analysis of olive oil and MCT oil formulations revealed that the lipid core’s physicochemical characteristics strongly influence NC formation, dispersity, and formulation robustness. Of the systems evaluated, MCT oil demonstrated superior colloidal behavior and greater reproducibility across the experimental domain, thus supporting its suitability as a lipid core for stable CEE-loaded polymeric NCs.

### 3.3. Physicochemical Characterization and Surface Morphology of Nanocapsules (NCs)

Dynamic light scattering (DLS) analysis (Figure 4) showed that both olive oil- and MCT oil-based NCs had a narrow hydrodynamic diameter distribution and high colloidal stability. Olive oil NCs had a hydrodynamic diameter of (231.2 ± 3.1) nm, a PDI of 0.196 ± 0.022, and a zeta potential close to +33 mV. By contrast, MCT oil NCs had a slightly smaller hydrodynamic diameter (206.9 ± 2.1) nm, PDI 0.130 ± 0.027, and zeta potential values around +31 mV.

The lower hydrodynamic diameter and dispersity observed for MCT oil systems may be attributed to the reduced viscosity and improved emulsification capacity of medium-chain triglycerides, which facilitate smaller droplet formation during solvent displacement. Similar effects have previously been described in lipid-based nanosystems containing MCT-rich cores [4].

Importantly, according to the number distribution analysis, more than 90% of the particle population in both systems remained below 200 nm (Figure 4B). Such NCs are considered advantageous for biological interactions and potential transport across physiological barriers [40].

The positive zeta potential observed in all formulations arises from protonated amino groups in CS. These surface charges contribute to electrostatic stabilization by generating repulsive interactions between particles and preventing aggregation during storage [16].

Scanning electron microscopy (SEM) analysis (Figure 5) confirmed spherical nanostructures with rounded edges and homogeneous morphology. SEM images show particle diameters between 150 and 170 nm, measured with microscopy software, slightly smaller than those obtained by DLS because the samples were dehydrated during electron microscopy analysis.

Surface analysis revealed smooth, compact exteriors in both formulations, with no evidence of crystalline aggregates or polymer collapse. However, subtle differences between the two systems were evident. Olive oil NCs had slightly rougher surfaces and more heterogeneity, whereas MCT oil NCs were more homogeneous and had a more regular spherical structure. These findings suggest that MCT oil enables more uniform interfacial deposition of CS during NC formation and are consistent with the response surface predictions, further supporting the influence of lipid core composition on NC assembly and interfacial organization [41].

Additionally, SEM images showed that the polymeric shell remained intact and continuous after solvent evaporation, indicating successful formation of stable core–shell architectures [9]. The absence of visible pores or surface fractures suggests that the lipid core is efficiently encapsulated, and that leakage of the encapsulated CEE is limited.

Taken together, the DLS and SEM results confirm that the developed systems have the appropriate nanoscale dimensions, surface homogeneity, and colloidal stability required for biomedical applications involving hydrophobic cannabinoids.

### 3.4. ATR-FTIR Analysis of Deconvoluted Spectra and Molecular Interactions

Figure 6 shows the ATR-FTIR spectra of CEE, empty NCs, and CEE-loaded NCs, and Table 1 summarizes the assignment of the main deconvoluted bands (full deconvolutions in the Supplementary Materials). Deconvolution improved the resolution of overlapping bands, particularly in the fingerprint region (1800–600 cm^−1^).

Pure CEE showed bands typical of cannabinoids and other phenolic phytocompounds [42]: aliphatic CH_2_ stretching (~2922 and 2859 cm^−1^), aromatic C=C stretching (~1628 and 1579 cm^−1^), and several C–O and C–O–C stretching bands between 1300 and 1000 cm^−1^ (Figure 6A). In the text, we refer to CBD because it is the major compound. Empty NCs showed the features of triglyceride-based systems stabilized by CS and Tween^®^ 80: the ester C=O stretching (~1740 cm^−1^), the aliphatic C–H stretching (~2924 and 2864 cm^−1^), and a broad O–H/N–H band (~3320 cm^−1^) from CS and Tween^®^ 80 (Figure 6B,C) [43].

After loading, both formulations retained the main bands of the carrier, and no new bands indicating covalent bond formation were detected, which suggests that CEE is incorporated through physical interactions rather than chemical modification, an advantage for preserving the structure of phytocannabinoids [44]. Shifts and intensity changes were observed in the O–H/N–H (~3330 cm^−1^), ester C=O (~1740 cm^−1^), and aromatic (~1600 cm^−1^) bands, and aromatic bands around 1590–1625 cm^−1^ appeared or were enhanced, consistent with the incorporation of CEE into the NCs [45]. The O–H/N–H band was more intense after loading, especially in olive oil NCs (Figure 6E), which suggests stronger hydrogen bonding between cannabinoid hydroxyl groups and CS amino groups [46].

MCT oil NCs showed sharper CH_2_ stretching bands (~2920–2850 cm^−1^), suggesting more ordered lipid packing, whereas olive oil NCs showed broader bands and slight shifts in the carbonyl region (~1739 cm^−1^), consistent with the greater fluidity and unsaturation of olive oil triglycerides [47] (Figure 6D,E). These differences could influence where CBD is located. Because CBD has both hydrophobic and phenolic hydroxyl groups, it may be located deeper in the core in the more ordered MCT oil, as suggested by the lower O–H/N–H intensity, and closer to the polymer–lipid interface in olive oil, where its hydroxyl groups can interact with CS and Tween^®^ 80, as suggested by the stronger, broader ~3330 cm^−1^ band and the stronger aromatic contribution at ~1600 cm^−1^ [48] (Figure 7). Since this interpretation rests on indirect spectroscopic evidence, it is a working hypothesis, which was further examined by confocal microscopy (Section 3.5.6).

### 3.5. Encapsulation Efficiency and Role of the Polymeric Matrix

#### 3.5.1. Encapsulation Efficiency

Both formulations showed high encapsulation efficiency, with more than 85% of the CBD added being recovered from the NCs after removal of the non-encapsulated fraction. These results demonstrate the suitability of CS-based polymeric NCs for encapsulating highly hydrophobic phytocannabinoids. Because a single extract batch was used, the encapsulation efficiency and stability data refer to this batch; whether batch-to-batch differences in CBD content or in minor cannabinoids and terpenes affect loading and stability remains to be evaluated.

This can be attributed to the NCs’ core–shell architecture, in which the hydrophobic lipid nucleus acts as a phytocannabinoid reservoir and the polymeric shell reduces diffusion into the external aqueous phase. Similar encapsulation efficiencies have been reported for cannabinoid-loaded nanocarriers prepared using lipid–polymer hybrid systems [4].

Although both lipid systems exhibited comparable encapsulation efficiencies, subsequent stability studies revealed differences in CBD preservation over time. MCT oil formulations retained CBD better than olive oil systems under both long-term and accelerated conditions.

These findings suggest that the polymeric shell primarily governs structural and colloidal stabilization, while the internal lipid phase strongly influences the chemical stabilization of the encapsulated phytocannabinoid. The antioxidant environment and lower susceptibility to oxidative degradation in MCT-rich systems may improve CBD preservation.

Therefore, the results emphasize the dual contribution of the NCs components: CS provides electrostatic and structural stability, while the lipid core determines the chemical microenvironment surrounding the encapsulated phytocannabinoids.

#### 3.5.2. Chemical Stability of CBD and Role of the Lipid Core

The chemical stability of CBD within the nanocapsules was evaluated under long-term and accelerated conditions through quantification of residual CBD content over time (Figure 8). All systems showed progressive CBD degradation [49] but substantial differences emerged between formulations prepared with MCT oil and olive oil.

MCT oil NCs consistently preserved CBD better than olive oil systems, particularly under elevated temperature and UV exposure. This behavior can be attributed to MCT’s lower oxidative susceptibility relative to the highly unsaturated fatty acids in olive oil [50]. Similar effects have been reported in lipid-based delivery systems, where unsaturated lipid matrices promote oxidative degradation of encapsulated compounds through free radical propagation mechanisms [4,51].

The ATR-FTIR results provide additional molecular-level insight into these differences. In MCT oil nanocapsules, the sharper CH_2_ stretching bands and lower O–H/N–H intensity suggest that CBD is preferentially localized deeper within the hydrophobic lipid core, where it is protected from environmental oxygen and moisture. This core-entrapment model is consistent with the experimentally observed enhanced chemical stability.

In contrast, olive oil nanocapsules exhibited stronger and broader O–H/N–H bands (~3330 cm^−1^) and enhanced aromatic signals around ~1600 cm^−1^ (Figure 6E), suggesting greater interfacial localization of CBD. In this configuration, phenolic hydroxyl groups of CBD remain partially exposed near the polymer–lipid interface, increasing their susceptibility to oxidation and environmental degradation.

Furthermore, slight shifts in the ester carbonyl region (~1736–1739 cm^−1^) suggest dipolar interactions between CBD and the lipid matrix. The more fluid, unsaturated environment of olive oil likely increases CBD mobility, which may enhance oxygen diffusion into the lipid core and accelerate degradation kinetics.

The preservation of the principal FTIR bands after storage additionally indicates that CBD degradation occurs mainly through partial oxidative processes rather than complete structural disruption of the nanocapsules. Importantly, no new absorption bands indicative of covalent degradation products or polymeric destabilization were observed, confirming that the nanocarrier architecture remained structurally stable despite partial phytocannabinoid degradation.

These findings reinforce the critical role of the lipid core composition in determining not only encapsulation efficiency but also long-term chemical protection of hydrophobic phytocannabinoids.

#### 3.5.3. Influence of pH on Nanocapsule (NC) Stability and Structural Organization

The influence of pH on NC stability was evaluated under physiologically relevant conditions (pH 5.5, 6.8, and 7.4) by monitoring changes in hydrodynamic diameter, zeta potential, and CBD retention (see Figure 9A–E).

Increasing pH progressively reduced zeta potential by deprotonating amino groups in CS. This decrease in positive surface charge was accompanied by moderate increases in hydrodynamic diameter, particularly at pH 7.4, indicating a partial reduction in electrostatic repulsion between particles.

Nevertheless, both MCT oil and olive oil NCs maintained nanometric dimensions, with no evidence of macroscopic aggregation or phase separation, thus demonstrating the preservation of their colloidal integrity. Similar pH-dependent behavior has been reported for CS-coated nanosystems, where electrostatic stabilization progressively decreases under neutral and alkaline conditions [19].

The stability observed under varying pH conditions correlates directly with the inferred intermolecular organization determined by ATR-FTIR analysis. The extensive hydrogen-bonding network indicated by the broad O–H/N–H band likely helps maintain structural cohesion, even as electrostatic stabilization decreases. Therefore, electrostatic and hydrogen-bond-mediated interactions appear to cooperate in preserving NCs integrity.

Notably, CBD remained associated with the NCs throughout the experiments, and no diffusion through the Franz membranes was detected (Figure 9D). This result suggests that the cannabinoids are strongly retained within the lipid–polymer matrix.

The stronger hydrophobic interactions inferred for MCT oil NCs may enhance CBD retention under acidic conditions. Conversely, the greater interfacial localization inferred for olive oil systems may allow for slightly higher molecular mobility, although no significant release was detected in experiments.

These findings suggest that NCs are robust enough to preserve their cannabinoids under conditions that simulate physiological environments.

#### 3.5.4. Freeze-Drying Stability and Structural Preservation

Studies on freeze-drying demonstrated that sucrose effectively protected the NCs during lyophilization and reconstitution. Rapid freezing in liquid nitrogen with 10% sucrose best preserved hydrodynamic diameter and PDI after redispersion: olive oil NCs had a hydrodynamic diameter of (219.8 ± 29.9) nm, a PDI of 0.303 ± 0.029, and zeta potential values close to (+12.7 ± 0.8) mV. By contrast, MCT oil NCs had a hydrodynamic diameter of (243.5 ± 8.5) nm, a PDI of 0.293 ± 0.016, and a zeta potential around (+16 ± 1) mV.

Although the zeta potential of the redispersed NCs remained positive, it was markedly lower than before lyophilization (+31 to +33 mV). This decrease may be explained by three non-exclusive factors: (i) the loss of volatile acetic acid during freeze-drying and the redispersion in ultrapure water, which raises the pH and reduces the protonation of the CS amino groups, in line with the pH dependence described in Section 3.5.3; (ii) the presence of non-ionic sucrose around the NCs, which can screen the surface charge and shift the slipping plane; and (iii) possible changes in polymer coverage during freezing and drying. The lower surface charge may also contribute to the higher PDI (≈0.3) observed after redispersion.

Scanning electron microscopy (SEM) analysis revealed that the NCs retained their spherical morphology after freeze-drying, with no evidence of severe aggregation or shell collapse (Figure 10). Sucrose’s cryoprotective effect is associated with forming a glassy matrix around the nanoparticles, which minimizes mechanical stress during ice crystal formation. Similar stabilization mechanisms have been described for polymeric and lipid nanocarriers subjected to lyophilization [33].

ATR-FTIR spectra of freeze-dried samples retained the main structural bands associated with CS, triglycerides, and cannabinoids. This indicates that lyophilization did not significantly alter the molecular organization of the systems. The persistent broad O–H/N–H band further suggests that the hydrogen-bonding network responsible for structural stabilization was maintained.

Notably, MCT oil NCs redissolved slightly better than olive oil systems, likely because FTIR analysis suggested more ordered lipid organization. The tighter packing of medium-chain triglycerides may reduce structural deformation during water removal and facilitate restoring the original colloidal arrangement upon rehydration.

Preserved CBD content after prolonged freeze-dried storage confirms the suitability of these nanosystems for pharmaceutical applications requiring long-term stability.

#### 3.5.5. Structure–Stability Relationship in Polymeric Nanocapsules (NCs)

Combined physicochemical, morphological, and ATR-FTIR analyses reveal a strong relationship between molecular organization and NC stability (see Figure 7).

The CS shell primarily stabilizes the colloid through electrostatic repulsion and hydrogen bonding, while the lipid core governs the internal microenvironment around CBD and therefore strongly influences chemical stability [7].

Deconvoluted ATR-FTIR spectra showed that MCT oil NCs have a more organized hydrophobic structure, favoring deeper CBD incorporation within the lipid core (Figure 6D). This arrangement limits phytocannabinoids’ exposure to the environment and improves chemical stability under stress conditions.

In contrast, the more unsaturated and fluid olive oil matrix appears to promote partial localization of CBD at the interface, enhancing hydrogen-bonding interactions with the polymeric shell but also increasing susceptibility to oxidative degradation (Figure 6E).

Confocal microscopy confirmed that the CBD-reactive signal resides in the oil (lipid) phase of both systems and not in the chitosan or the aqueous medium (Figure 11), but its resolution does not allow discrimination between core and interfacial CBD within individual NCs; the formulation-dependent localization proposed here should therefore be considered a working hypothesis supported by the ATR-FTIR data (Figure 7), not a demonstrated structural feature (Section 3.5.6).

Therefore, the physicochemical stability of these NCs emerges from a synergistic interplay between [8,19]:

(i) Electrostatic stabilization provided by CS;
(ii) Hydrogen-bond-mediated structural cohesion;
(iii) Hydrophobic interactions between phytocannabinoids and triglycerides;
(iv) The molecular organization imposed by the lipid composition.

Overall, the results show that optimizing the internal lipid structure is as important as stabilizing the polymeric shell to develop efficient nanocarriers for phytocannabinoid delivery.

#### 3.5.6. Spatial Distribution of CBD Within the NCs: Confocal Microscopy with Fast Blue

To provide independent evidence of where CBD is located in the NCs, complementing the ATR-FTIR analysis (Section 3.4), CBD-loaded and empty NCs were labeled with Fast Blue and examined by confocal laser scanning microscopy (CLSM; Figure 11). CBD-loaded MCT oil and olive oil NCs appeared in bright fields as dense aggregates of spherical particles and showed intense fluorescence in both detection channels (Figure 11A,C), whereas empty NCs imaged under identical conditions showed only a weak, diffuse signal (Figure 11B,D). The 99th-percentile intensity of CBD-loaded NCs was 3.4- to 4.3-fold (515/30 nm) and 4.8- to 6.0-fold (605/75 nm) higher than that of the corresponding empty NCs and more than 100-fold higher than that of the blanks (chitosan solution, olive oil and MCT oil; Table S6 and Figure S6), while Fast Blue alone gave only a faint, sparse signal (Figure S6D). These controls indicate that the signal arises mainly from Fast Blue reacting with the encapsulated cannabinoids, not from the dye, the polymer, or the oils alone. The residual signal of the empty olive oil NCs, which varied among fields (Table S6), may be due to the reactivity of Fast Blue toward other phenolic compounds present in olive oil and to autofluorescence; it also indicates that Fast Blue is a CBD-reactive, rather than strictly CBD-specific, probe [32].

Within the loaded aggregates, the two channels showed different distributions (Figure 11A,C). The 605/75 nm (red) signal was concentrated in well-defined spherical domains of about 1–3 µm that coincided with the lipid droplets visible in bright field, whereas the 515/30 nm (green) signal was more diffuse throughout the aggregate. Therefore, in both formulations the CBD-reactive signal is associated with the oil phase and not with the chitosan or the aqueous medium, which is consistent with the high encapsulation efficiency (Section 3.5.1). The diffuse green component, however, does not allow a minor contribution from the polymer matrix to be completely excluded.

Taken together, the observations in Figure 11 indicate that Fast Blue reacts with CBD retained in the NC aggregates: the signal is intense only when the extract is present and is not reproduced by chitosan, the oils, or the dye alone. The red channel follows the lipid droplets, placing this CBD in the oil phase, consistent with the high encapsulation efficiency, whereas the diffuse green channel may reflect dye products dispersed through the aggregate. The residual signal of empty olive oil NCs is consistent with the reactivity of Fast Blue toward olive oil phenolics, and no clear difference between MCT oil and olive oil NCs was apparent. CLSM therefore supports oil-phase retention of CBD but not, by itself, the lipid-dependent localization proposed from ATR-FTIR.

Two limitations should be considered. First, the droplets resolved by CLSM (≥1 µm) are an order of magnitude larger than individual NCs (207–231 nm by DLS and 150–170 nm by SEM; Section 3.3) and probably correspond to NC aggregates or coalesced lipid domains formed on the slide; since the lateral resolution of the 60×/1.40 NA objective (~0.2–0.3 µm) is comparable to the NC diameter, CLSM cannot resolve the core–shell architecture of single NCs. As with any dye-based approach, the signal also reports the location of the labelled product, which may not coincide exactly with that of unreacted CBD [52]. Second, the images are single focal planes of a limited number of fields (Table S6), and volumetric (3D) imaging was not performed, so the comparison is descriptive.

The formulation-dependent localization suggested by the ATR-FTIR deconvolution (Section 3.4 and Figure 7) should therefore be regarded as a working hypothesis, to be confirmed by complementary techniques.

### 3.6. Antiviral Activity

The antiviral activity of the formulations against CHIKV was evaluated by measuring the inhibition of virus-induced CPE using the NR uptake assay. Dose–response profiles are shown in Supplementary Figure S7. CPE inhibition and viability were compared against the virus and formulation controls using Dunnett’s test, and samples were compared using two-way ANOVA (sample × concentration) followed by Holm-adjusted comparisons at each concentration. Antiviral activity was evaluated at CBD concentrations associated with cell viability above 70% (Table 2).

Since the CEE-loaded formulations did not reach 50% CPE inhibition within the evaluated concentration range compatible with the predefined cell viability threshold, EC_50_ values could not be determined, and selectivity indices could not be calculated.

CEE showed antiviral activity against CHIKV in the CPE inhibition assay, although its activity was observed within a narrow window between inhibition and cytotoxicity. Free CEE in olive oil showed the highest inhibition (71.3% at 0.167 mg.mL^−1^). The CEE-loaded NCs achieved up to approximately 34–45% CPE inhibition, significantly exceeding that of the corresponding empty NCs at the evaluated intermediate concentrations. Empty NCs generally showed negligible inhibition. These findings support the contribution of the incorporated CEE to the observed inhibitory effect. Thus, these results do not show that nanoencapsulation improves CEE selectivity or therapeutic window against CHIKV.

Free CEE formulations showed higher CPE inhibition in olive oil than in MCT (71.3% vs. 35.6%), whereas CEE-loaded NCs showed broadly comparable inhibition (44.1% vs. 34.1%). Therefore, these findings are preliminary. Confirmation using an orthogonal assay, such as infectious viral titer determination, is also needed to establish whether the observed reduction in CPE reflects inhibition of viral replication. RT-qPCR could provide complementary information on viral RNA levels. These studies will clarify nanoencapsulation’s contribution to antiviral activity and determine whether it improves CEE selectivity against CHIKV.

## 4. Limitations of the Study

Several limitations should be considered when interpreting this study’s results. First, all formulations were prepared from a single batch of ethanolic *Cannabis sativa* extract obtained from a defined, CBD-rich chemotype. While this approach reduced variability associated with the botanical source, cannabis extract composition can still vary with cultivation, harvesting, and extraction conditions. Therefore, batch-to-batch variability was not assessed, and the reproducibility of the observed formulation properties with independent extract batches and their transferability to extracts with different cannabinoid and terpene profiles have yet to be established. In addition, encapsulation efficiency and stability were quantified for CBD only, and the behavior of minor cannabinoids and terpenes was not assessed. Nevertheless, the study prepared the nanocapsules repeatedly using the same procedure, and the physicochemical properties of the optimized formulations were consistent across preparations.

Second, the polymer selection considered chitosan molecular weight and degree of deacetylation; however, different chitosan grades were not experimentally compared. Consequently, the individual contributions of these properties, as well as those of viscosity and acetyl group distribution, to nanocapsule formation and colloidal stability could not be determined. Further studies using chitosan with systematically varied and fully characterized properties would help to clarify these relationships.

Third, while scanning electron microscopy (SEM), attenuated total reflectance Fourier-transform infrared spectroscopy (ATR-FTIR), and confocal fluorescence microscopy provided complementary information on nanocapsule morphology, chemical interactions, and the distribution of the CBD-reactive signal, these techniques do not directly establish CBD’s precise location within the nanocapsules. Confocal microscopy could not distinguish between localization in the lipid core and at the polymer–lipid interface, nor was any volumetric imaging performed. Therefore, the suggested differences in CBD localization between MCT oil and olive oil nanocapsules should be treated as hypotheses requiring confirmation with additional structural techniques.

Finally, the biological evaluation was limited to a single cell line (Vero), one CHIKV strain, and one multiplicity of infection, and relied on an indirect CPE readout based on neutral red uptake, without a reference antiviral as a positive control. Because the formulations were added at the time of infection, the stage or mechanism of inhibition could not be determined. CEE-loaded NCs showed only modest inhibition (34.1% for MCT oil NCs and 44.1% for olive oil NCs at concentrations that maintained cell viability above 70%), and the narrow window between inhibition and cytotoxicity prevented determination of EC_50_ values and selectivity indices. Free CEE in oil showed equal or higher inhibition (35.6% in MCT oil and 71.3% in olive oil), although a higher concentration of CBD was tested in the free-CBD formulation (0.167–0.250 mg/mL) compared to the NC formulations (0.059–0.061 mg/mL). To establish the antiviral potential of these formulations, orthogonal assays (infectious viral titer, RT-qPCR), time-of-addition experiments, additional cell types, and a comparison with an equivalent non-encapsulated dispersion are required.

Overall, these limitations mean the current study is a proof of concept for formulation and physicochemical characterization, with only preliminary in vitro biological evidence. Further analytical, structural, reproducibility, and biological studies are needed before drawing definitive conclusions about the molecular organization of CBD within the nanocapsules, the platform’s generalizability to other cannabis extracts, and its potential therapeutic performance.

## 5. Conclusions

This study demonstrates the feasibility of chitosan-based polymeric lipid nanocapsules for encapsulating a *Cannabis sativa* ethanolic extract, yielding colloidally stable, positively charged nanometric systems with high CBD encapsulation efficiency. The lipid core influenced nanocapsule size and homogeneity, favoring MCT oil, whereas CBD stability depended mainly on the oil vehicle itself; both systems retained CBD between pH 5.5 and 7.4 and could be redispersed after freeze-drying. ATR-FTIR and confocal microscopy indicated that CBD resides in the oil phase, although its core or interfacial position could not be resolved. In a preliminary antiviral screening against chikungunya virus, the nanocapsules showed modest inhibition of the cytopathic effect, with no advantage over the free extract. Future work should assess independent extract batches, the contribution of the polymeric shell, structural techniques to locate CBD, and orthogonal antiviral assays.

## Figures and Tables

**Figure 1:**
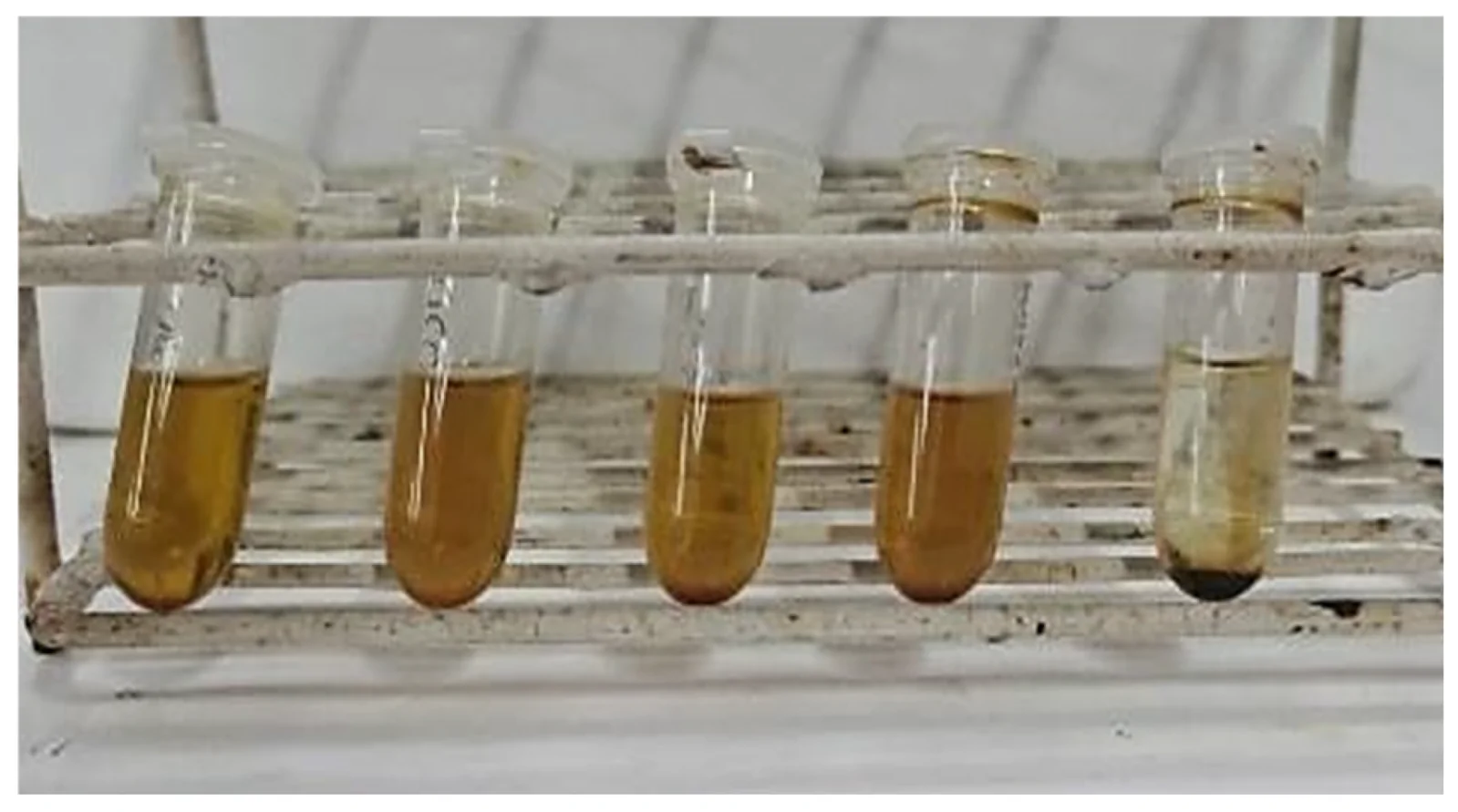
Solubility test of the CEE in different vegetable oils (from left to right: olive, MCT, sunflower, corn, and castor oil). Only olive oil and MCT oil fully solubilize the CEE without chromatographic interference or phase separation.

**Figure 2:**
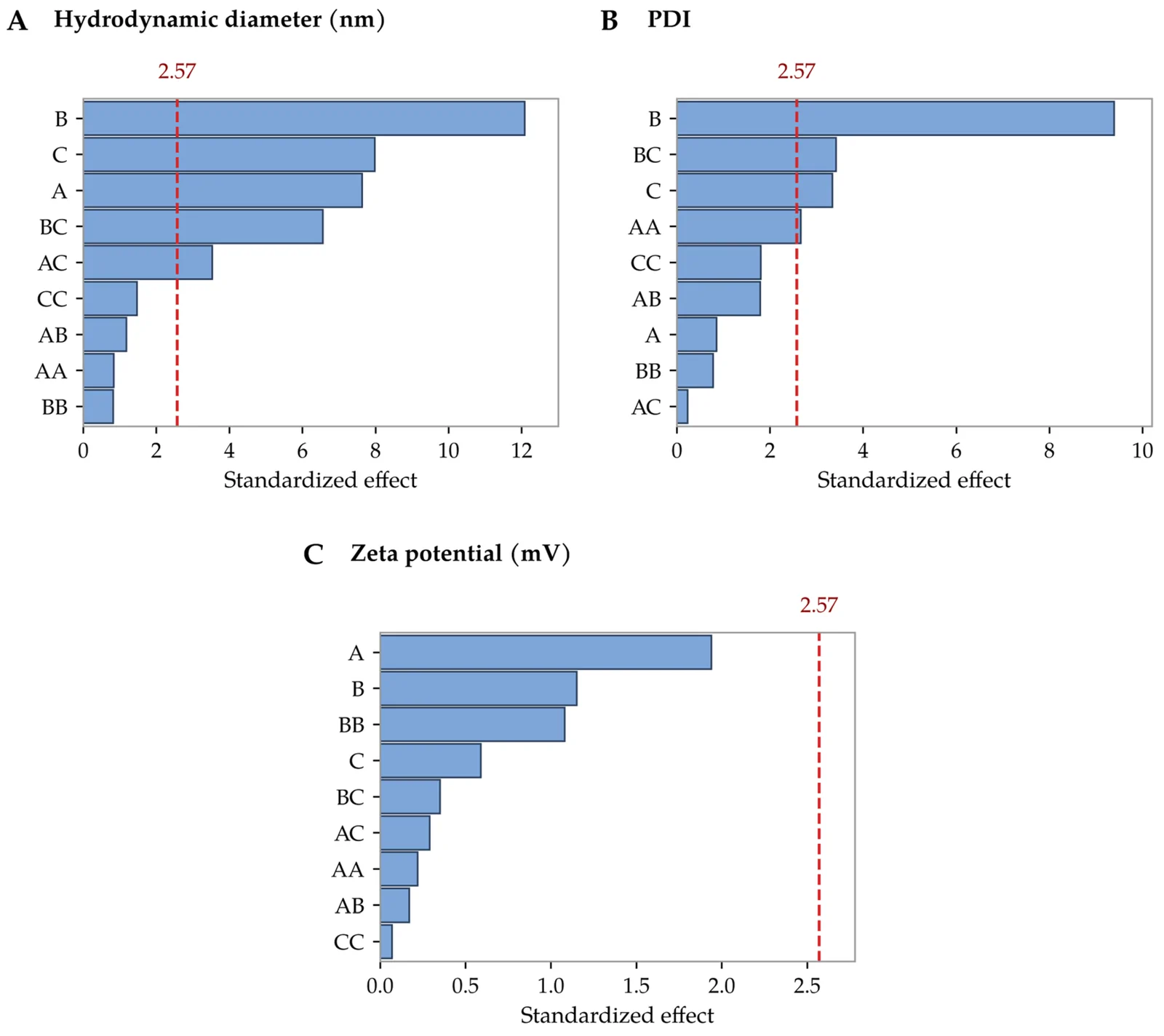
Pareto charts of the standardized effects of the formulation variables on (**A**) hydrodynamic diameter (nm), (**B**) polydispersity index (PDI), and (**C**) zeta potential (mV) of olive oil nanocapsules (Box–Behnken design, full quadratic model). A, CS concentration; B, oil volume; C, Tween^®^ 80 volume; AA, BB and CC, quadratic terms; AB, AC and BC, two-factor interactions. The dashed line indicates the significance threshold (α = 0.05; t = 2.57).

**Figure 3:**
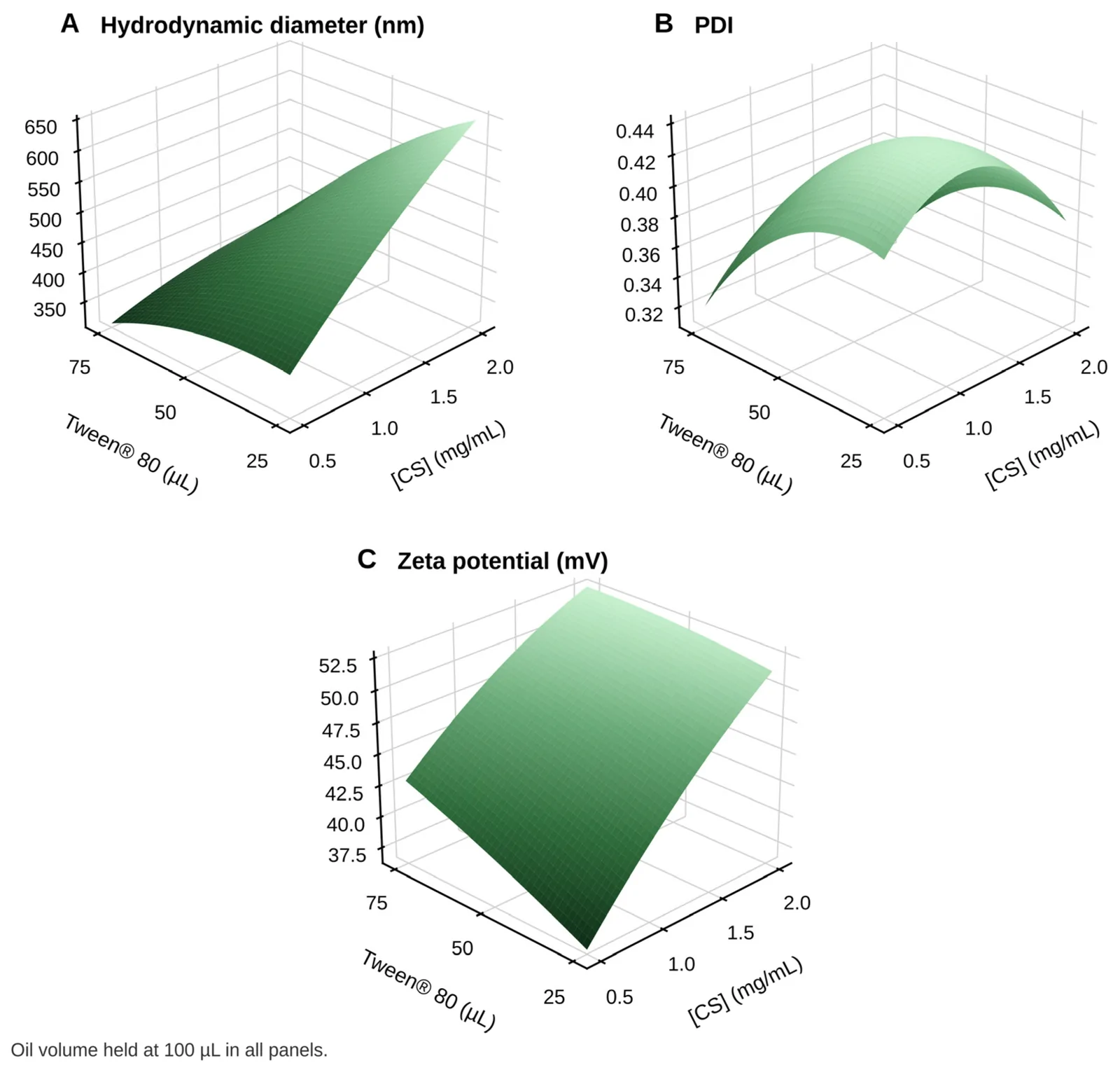
Response surface plots of (**A**) hydrodynamic diameter (nm), (**B**) polydispersity index (PDI), and (**C**) zeta potential (mV) as a function of CS concentration and Tween^®^ 80 volume, with oil volume held at 100 µL (Box–Behnken design).

**Figure 4:**
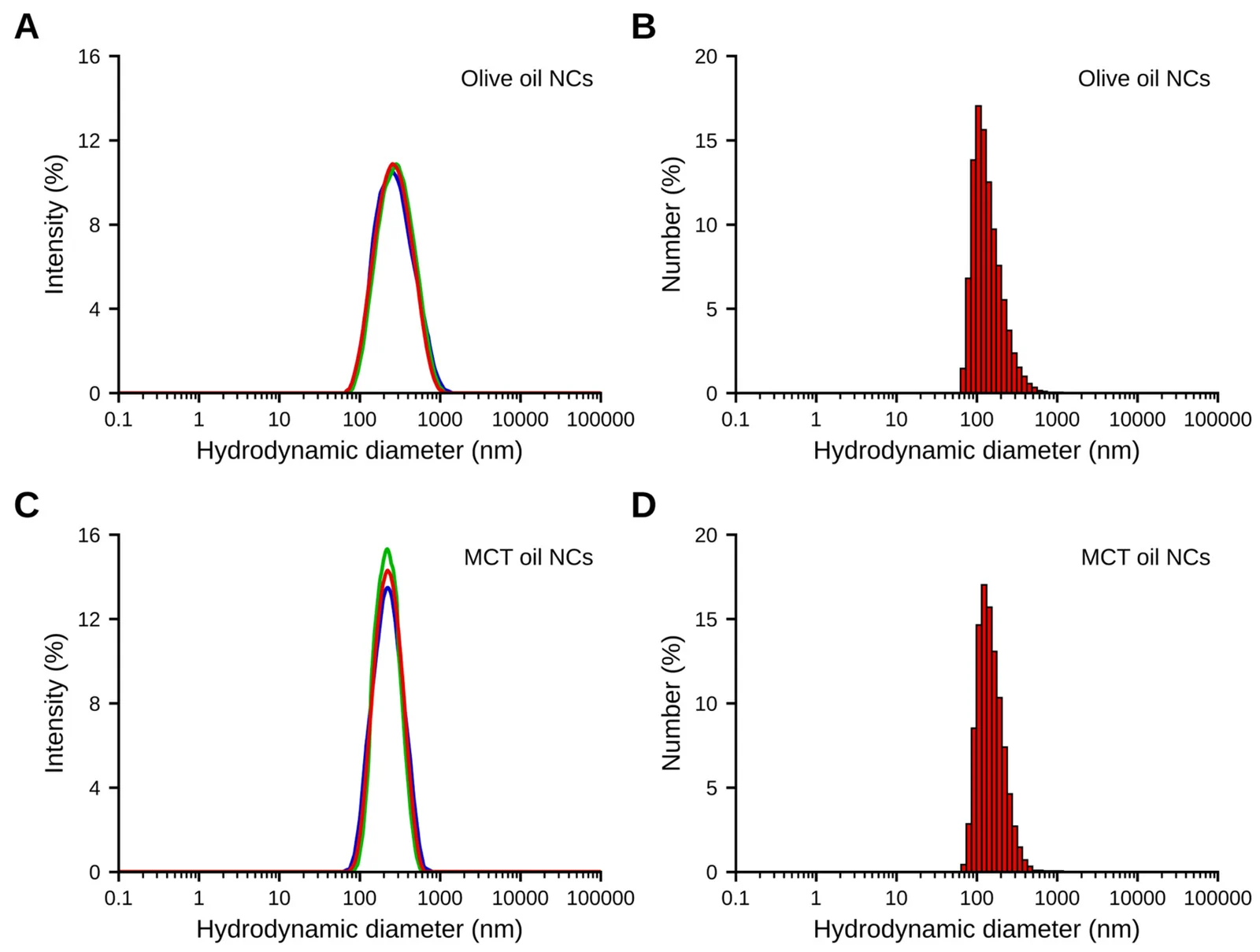
Hydrodynamic diameter distributions by dynamic light scattering (DLS). Intensity-weighted (**A**,**C**) and number-weighted (**B**,**D**) distributions for olive oil NCs (**A**,**B**) and MCT oil NCs (**C**,**D**). The three curves in A and C correspond to replicate measurements.

**Figure 5:**
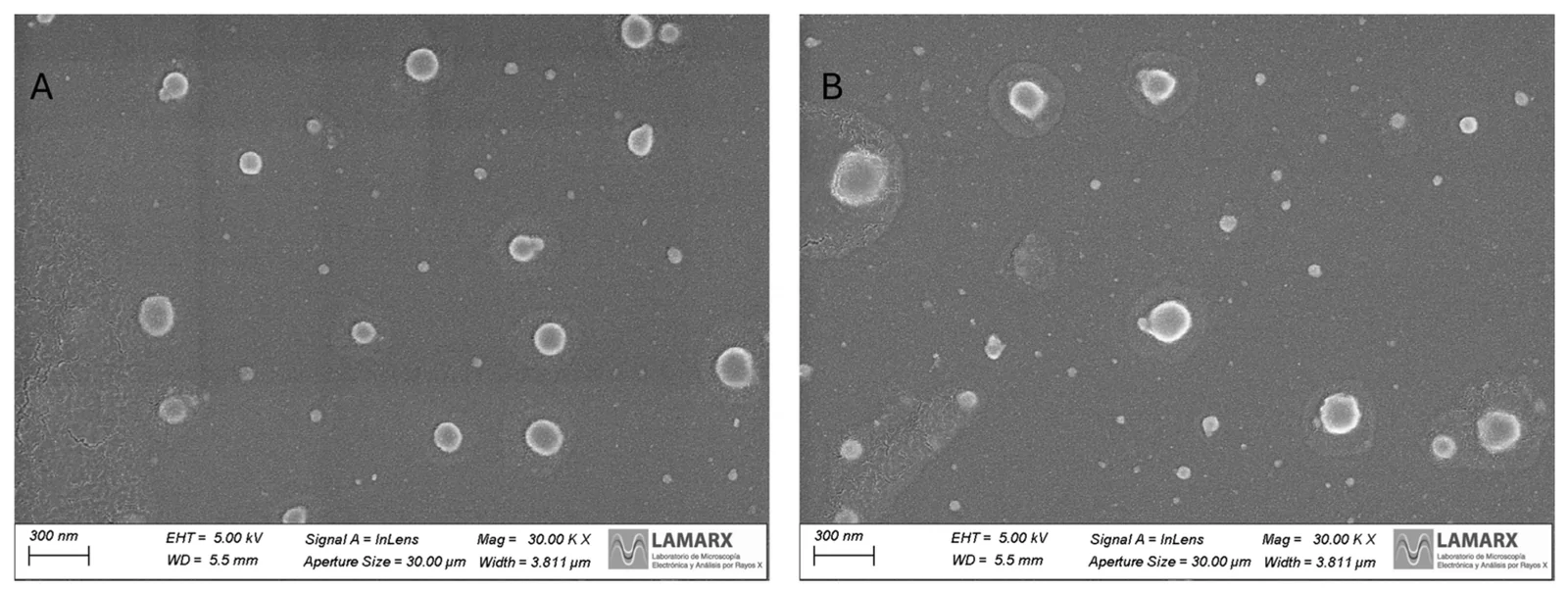
Scanning electron microscopy (SEM) images of (**A**) olive oil NCs and (**B**) MCT oil NCs.

**Figure 6:**
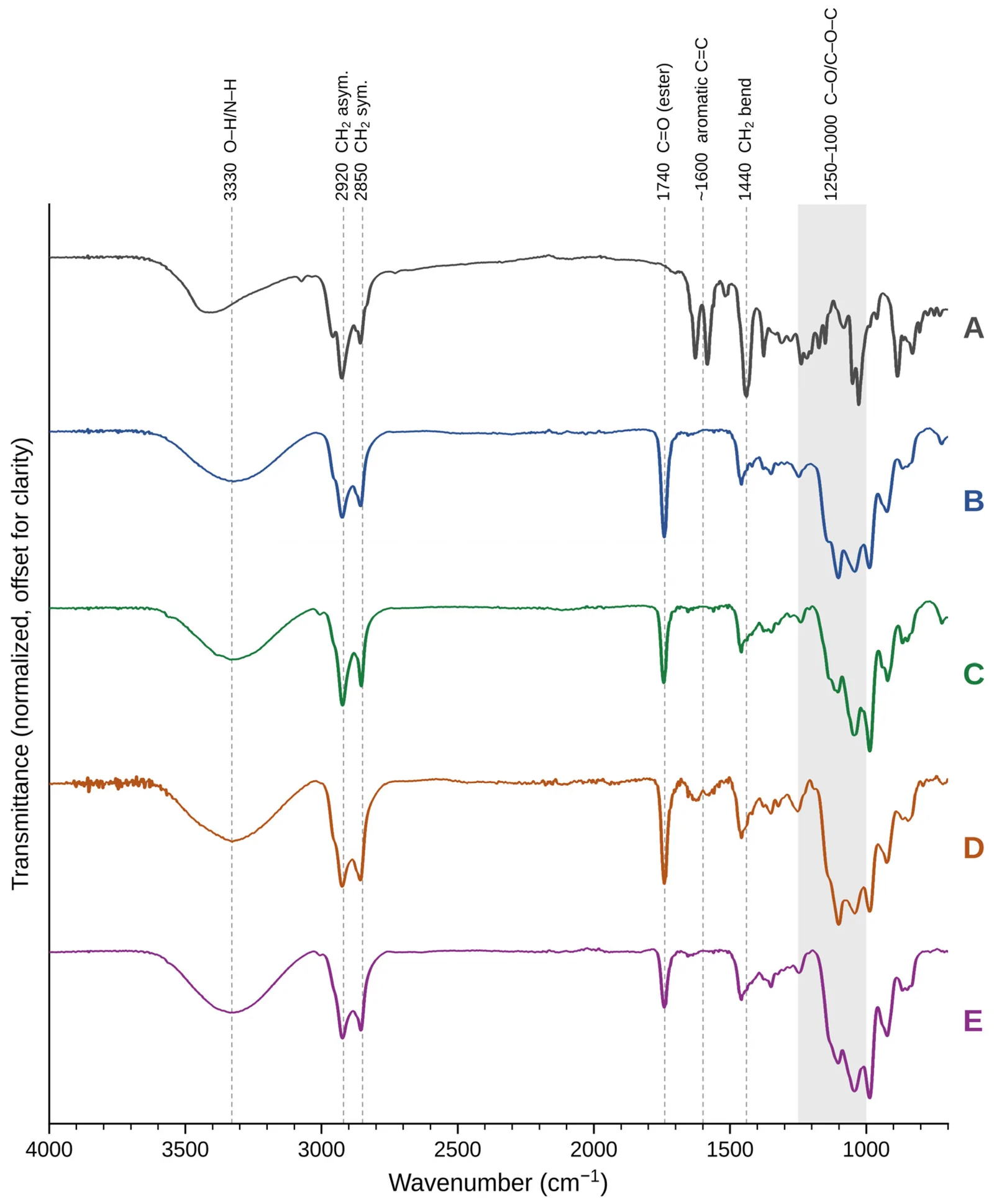
ATR-FTIR spectra of the CEE and chitosan-coated lipid-core nanocapsules (NCs). (**A**) CEE; (**B**) empty MCT oil NCs; (**C**) empty olive oil NCs; (**D**) CEE-loaded MCT oil NCs; (**E**) CEE-loaded olive oil NCs. Spectra are shown as %Transmittance, individually normalized and vertically offset for clarity of comparison.

**Figure 7:**
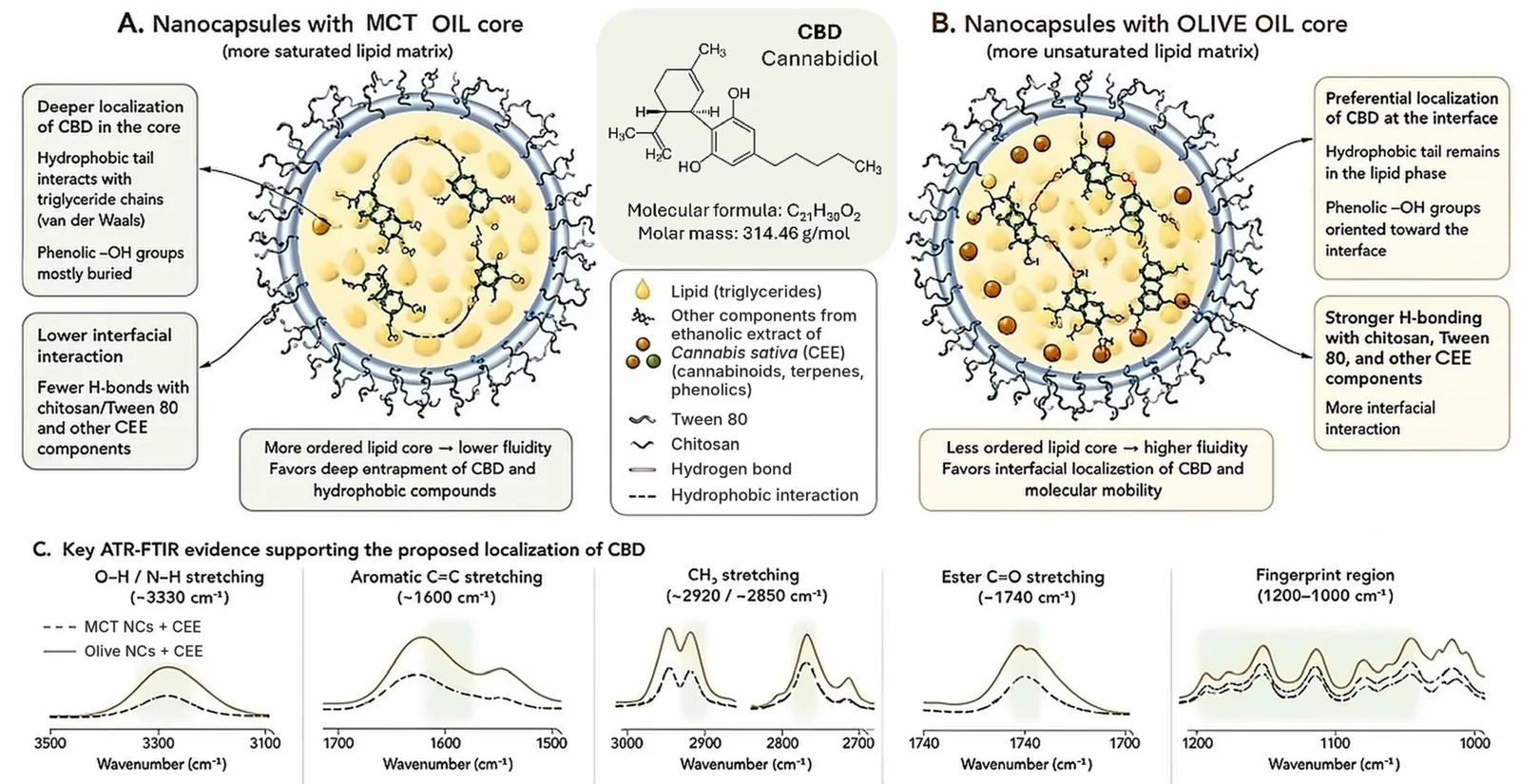
Proposed model of the localization and molecular interactions of cannabidiol (CBD) in nanocapsules (NCs) with MCT oil and olive oil cores, and key ATR-FTIR evidence (schematic representation). (**A**) NCs with an MCT oil core (more saturated lipid matrix), in which the more ordered lipid matrix is proposed to promote deeper entrapment of CBD in the core. (**B**) NCs with an olive oil core (more unsaturated, fluid lipid matrix), in which CBD is proposed to localize preferentially at the polymer–lipid interface. (**C**) ATR-FTIR spectra of MCT oil NCs + CEE (dashed line) and olive oil NCs + CEE (solid line) in the key vibrational regions: the O–H/N–H stretching (~3330 cm^−1^) suggests increased hydrogen bonding in olive oil NCs; the aromatic C=C stretching (~1600 cm^−1^) and the CH_2_ stretching (~2920/2850 cm^−1^) suggest differences in lipid packing and CBD environment; and the ester C=O stretching (~1740 cm^−1^) and the fingerprint region (1200–1000 cm^−1^) suggest distinct dipolar interactions and a higher contribution of CBD–polymer–lipid interactions in the olive oil-based systems. This localization is inferred from indirect spectroscopic evidence and was not demonstrated structurally (see Section 3.5.6 and Section 4). Note: The schematic diagram in this figure was created with the aid of generative artificial intelligence (Gemini, 4 Argon, Google). The authors reviewed the content and take full responsibility for its scientific accuracy.

**Figure 8:**
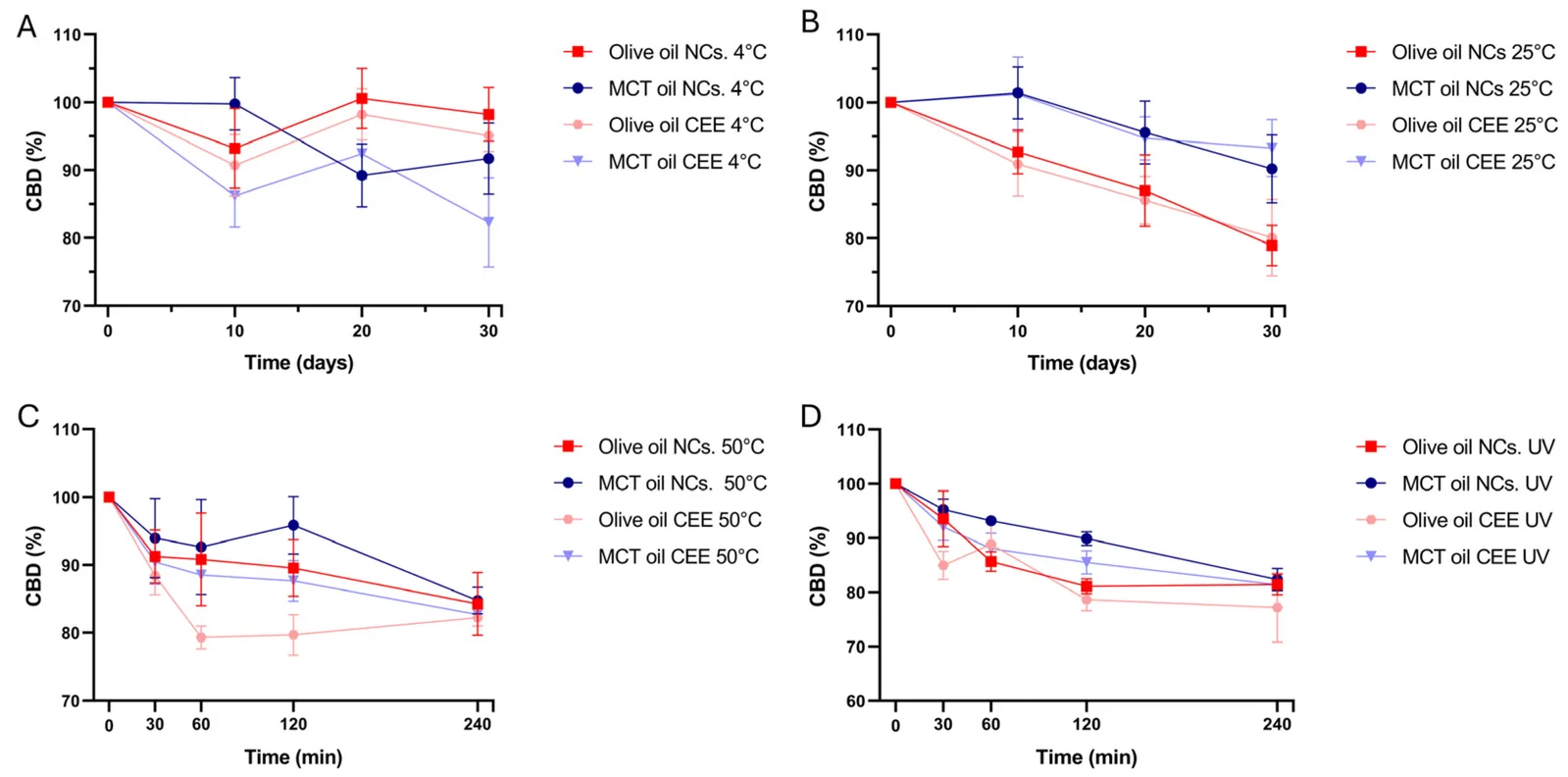
Evolution of residual CBD content (%) in olive oil NCs and MCT oil NCs, and in the corresponding CBD-containing oils, under long-term storage at 4 °C (**A**) and room temperature (**B**), and under forced stress at 50 °C (**C**) and UV exposure (**D**). Data are the mean ± SD (n = 3).

**Figure 9:**
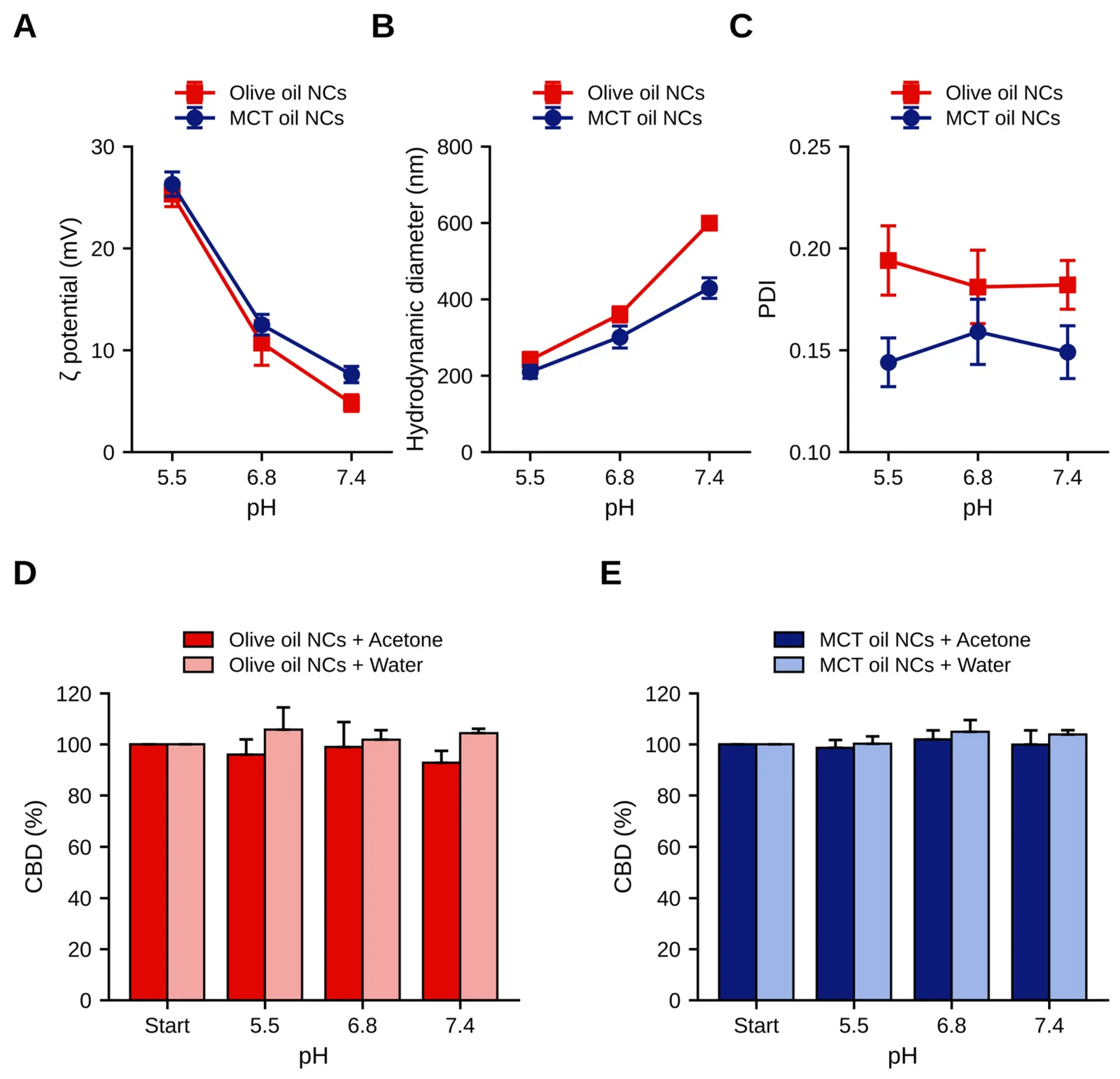
Influence of pH on nanocapsule (NC) properties and CBD retention. Zeta potential (**A**), hydrodynamic diameter (**B**), and PDI (**C**) of olive oil and MCT oil NCs at pH 5.5, 6.8, and 7.4. Percentage of CBD quantified in water (donor compartment, intact NCs) versus acetone (total content, disrupted NCs) for olive oil NCs (**D**) and MCT oil NCs (**E**), confirming that CBD remained fully associated with the NCs, with no diffusion across the Franz membrane. Data are the mean ± SD (n = 3).

**Figure 10:**
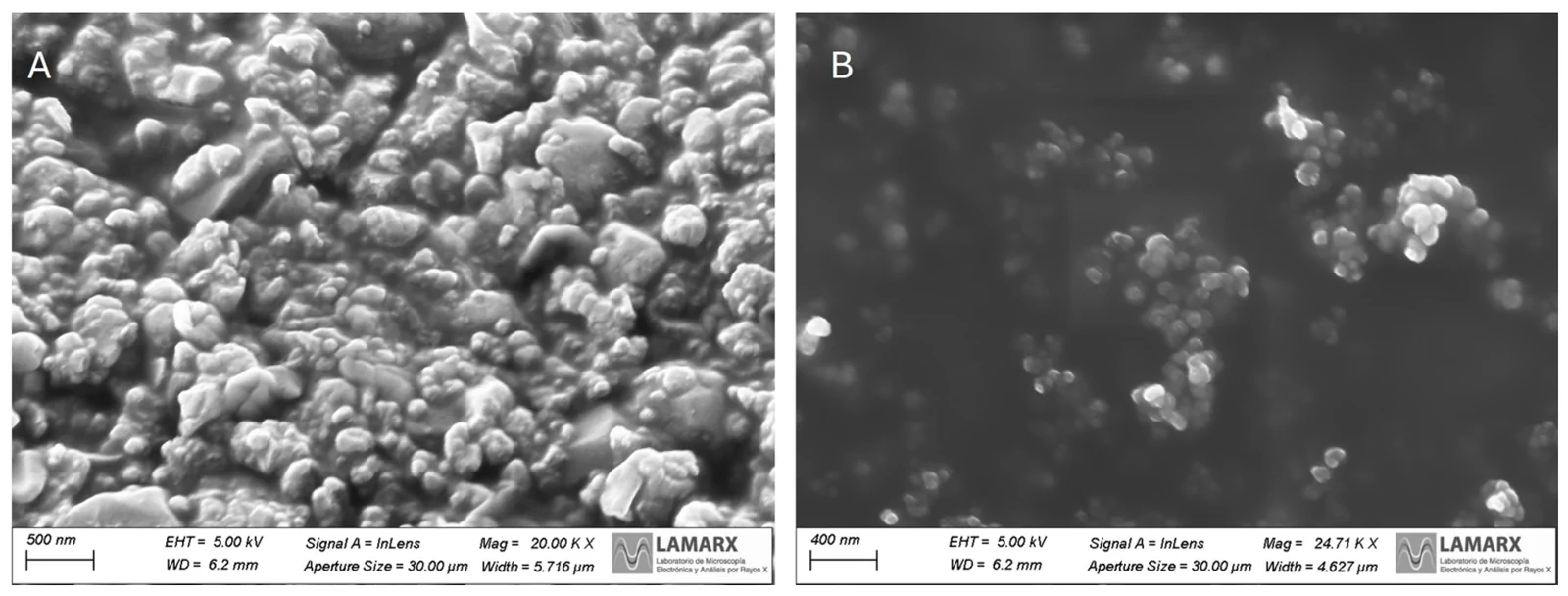
Scanning electron microscopy (SEM) images of freeze-dried (**A**) olive oil NCs and (**B**) MCT oil NCs, showing preserved spherical morphology embedded within the sucrose glassy matrix.

**Figure 11:**
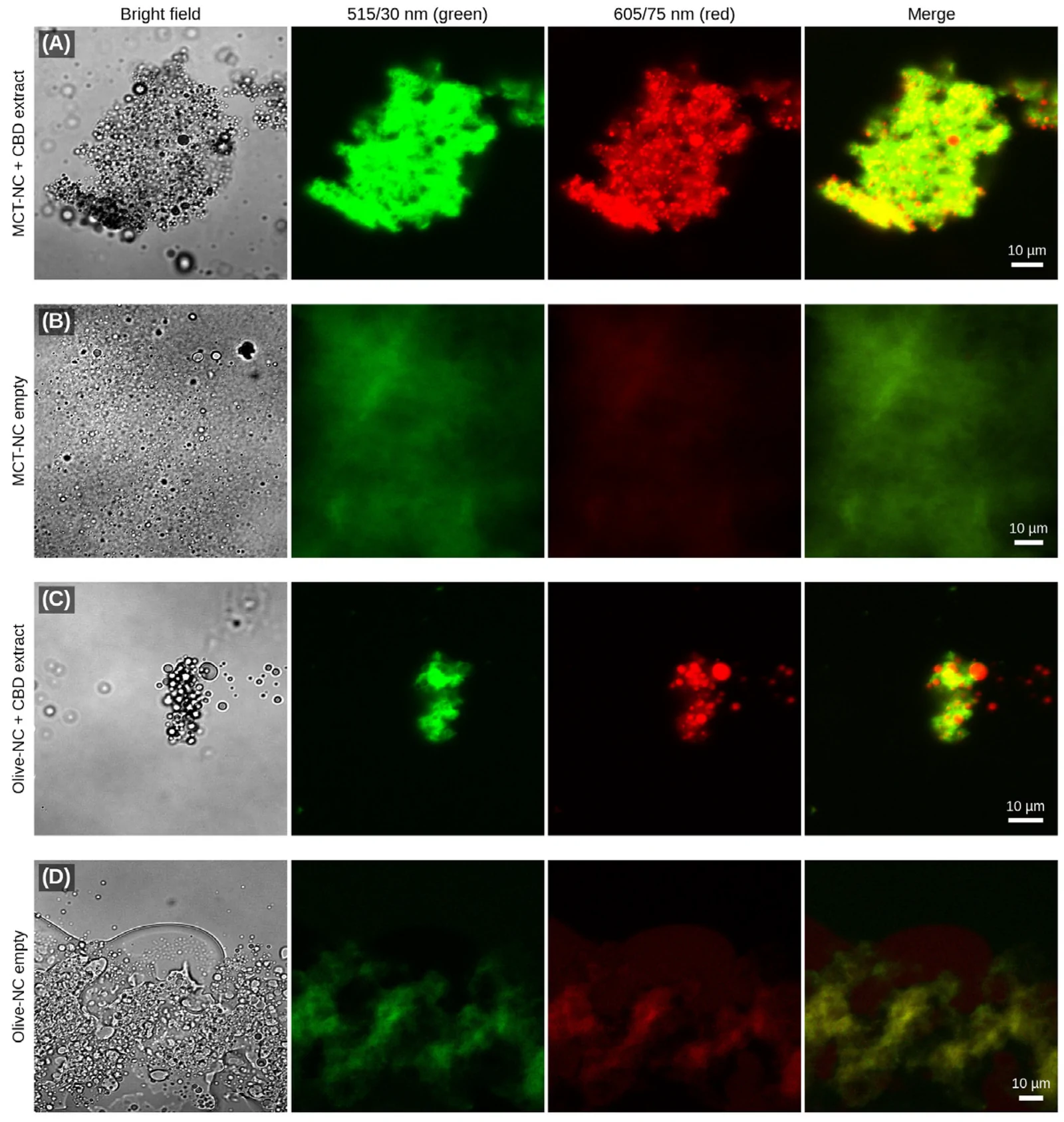
Confocal laser scanning microscopy (CLSM) of Fast Blue-labelled NCs. (**A**) CBD-loaded MCT oil NCs, (**B**) empty MCT oil NCs, (**C**) CBD-loaded olive oil NCs, and (**D**) empty olive oil NCs. Columns show the bright-field image, the 515/30 nm (green) and 605/75 nm (red) emission channels (488 nm excitation), and their merge. All fluorescence images were acquired with the same laser power and similar detector gain and are displayed with identical intensity scaling (20–2200 ADU for 515/30 nm and 20–1600 ADU for 605/75 nm); bright-field contrast was adjusted individually. Panel (**D**) is a 512 × 512-pixel crop of the original field. Scale bars: 10 µm.

**Table 1:** Assignment of the main deconvoluted ATR-FTIR bands for pure CEE, empty nanocapsules (NCs), and CEE-loaded NCs prepared with MCT oil or olive oil.

Wavenumber (cm^−1^)	Pure CEE	Empty MCT NCs	Empty Olive Oil NCs	Loaded MCT NCs	Loaded Olive Oil NCs	Functional Group Assignment	Main Contribution/Interpretation
~3330–3320	–	3319	3320	3330	3330	O–H/N–H stretching	Hydrogen bonding involving chitosan amino groups and phenolic hydroxyl groups from cannabinoids; increased intensity after loading indicates intermolecular interactions
~2925–2915	2922	2924	2922	2915	2923	CH_2_ asymmetric stretching	Aliphatic chains from triglycerides and cannabinoid structures
~2865–2855	2859	2864	2858	2859	2862	CH_2_ symmetric stretching	Lipid core organization and hydrophobic chains
~1740–1735	1715 (weak)	1740	1742	1736	1739	Ester C=O stretching	Triglyceride ester groups; slight shifts after encapsulation indicate dipolar interactions between oils and phytocannabinoids
~1645–1625	1628	1625	1640	1624	1642	Aromatic C=C/amide I	Cannabinoid aromatic structures and chitosan contribution
~1595–1575	1579	–	1587	1590	1590	Aromatic C=C stretching	Evidence of phytocannabinoid incorporation, mainly CBD aromatic rings
~1455–1435	1439	1453	1436	1438	1440	CH_2_ bending vibrations	Lipid chain organization and overlap between oil and cannabinoid signals
~1375–1360	1373	1365	1370	1357	1371	CH_3_ bending	Aliphatic groups from triglycerides and cannabinoids
~1300–1275	1309–1277	1295–1247	1275	1280	1279	C–O stretching	Ester and phenolic group contributions
~1240–1210	1236–1210	1247	1221	1216	1220	C–O/C–O–C stretching	Chitosan, Tween 80, and phytocannabinoid overlap
~1165–1040	1160–1043	1110–1040	1122–1048	1105–1045	1115–1051	C–O–C/C–O stretching	Polysaccharide backbone, surfactant, and cannabinoid contributions
~990–960	986–968	988	994	989–958	992	=C–H bending/C–O	CEE-related vibrational modes
~900–800	884–836–799	927–844	937–896–855	882–845	904–853	Aromatic C–H out-of-plane bending	Cannabis-derived aromatic structures
~750–700	734–705	736–717	752–705	746–709	752–701	C–H bending	Lipid and aromatic skeletal contributions
~620–600	667	624	604	610	602	Skeletal vibrations	Complex fingerprint region of multicomponent nanostructured systems

**Table 2:** Cell viability and inhibition of CHIKV-induced cytopathic effect by CEE-loaded MCT oil NCs and CEE-loaded olive oil NCs at different CBD concentrations.

	CBD Concentration (mg.mL^−1^)	Cellular Viability (%)	CPE Inhibition (%) (*p*-Value) *
CEE-loaded MCT oil NC	0.059	73.6 ± 2.1	34.1 ± 2.4 (*p* < 0.0001)
Empty MCT oil NCs	0	89.5 ± 1.3	0.8 ± 1.4 (ns) **
CEE MCT oil	0.250	83.9 ± 2.8	35.6 ± 7.3 (*p* = 0.027)
CEE-loaded olive oil NCs	0.061	76.8 ± 1.2	44.1 ± 0.9 (*p* < 0.0001)
Empty olive oil NCs	0	99.9 ± 0.1	1.6 ± 0.2 (ns) **
CEE olive oil	0.167	76.1 ± 0.8	71.3 ± 1.3 (*p* = 0.0005)

* *p*-value: Dunnett’s test of infected treated wells versus the virus control. ** not significant. Note: CBD concentration, determined by HPLC, for the NCs and for the free CEE in oil.

## Data Availability

The original contributions presented in this study are included in the article. Further inquiries can be directed to the corresponding authors.

## Supplementary Materials

The following supporting information can be downloaded at: https://www.mdpi.com/article/10.3390/polym18212701/s1, Deconvoluted ATR-FTIR Spectra of the *Cannabis sativa* Ethanolic Extract and chitosan-coated lipid nanocapsules; Figure S1: Deconvoluted ATR-FTIR spectrum of the pure *Cannabis sativa* ethanolic extract (CEE); Table S1: Center position, height, and full width at half height (FWHH) of the individual bands resolved from the deconvoluted ATR-FTIR spectrum of the pure CEE; Figure S2: Deconvoluted ATR-FTIR spectrum of empty chitosan-coated MCT oil nanocapsules (NCs); Table S2: Center position, height, FWHH, and area of the individual bands resolved from the deconvoluted ATR-FTIR spectrum of the empty MCT oil NCs; Figure S3: Deconvoluted ATR-FTIR spectrum of CEE-loaded chitosan-coated MCT oil nanocapsules; Table S3: Center position, height, FWHH, and area of the individual bands resolved from the deconvoluted ATR-FTIR spectrum of the CEE-loaded MCT oil NCs; Figure S4: Deconvoluted ATR-FTIR spectrum of empty chitosan-coated olive oil nanocapsules; Table S4: Center position, height, FWHH, and area of the individual bands resolved from the deconvoluted ATR-FTIR spectrum of the empty olive oil NCs; Figure S5: Deconvoluted ATR-FTIR spectrum of CEE-loaded chitosan-coated olive oil nanocapsules; Table S5: Center position, height, FWHH, and area of the individual bands resolved from the deconvoluted ATR-FTIR spectrum of the CEE-loaded olive oil NCs. Figure S6: Confocal laser scanning microscopy of control samples and of additional fields of Fast Blue-labelled NCs; Table S6: Acquisition parameters and quantitative analysis of all confocal images. Figure S7. Concentration-dependent effects of CEE-loaded NCs, empty NCs and CEE oil on Vero cell viability and CHIKV-induced cytopathic effect.
