# Supplementary material for: Chitosan-Based Polymeric Lipid Nanocapsules Loaded with Cannabis sativa L. Extract: Influence of Lipid Core Composition on Physicochemical Stability and Preliminary Anti-Chikungunya Virus Activity
Source: Polymers (Basel). 2026 Oct 31;18(21):2701. doi: 10.3390/polym18212701 (PMC13765891; doi:10.3390/polym18212701)
Supplement: Supplementary file 1 [file polymers-18-02701-s001.zip › polymers-4632139-supplementary.pdf]

Chitosan-based polymeric lipid nanocapsules loaded with *Cannabis sativa* extract: influence of lipid core composition on physicochemical stability and preliminary anti-chikungunya virus activity

Supplementary Material:

Deconvoluted ATR-FTIR Spectra of the Cannabis sativa Ethanolic Extract and Chitosan-Coated Lipid Nanocapsules

This file presents deconvoluted attenuated total reflectance Fourier-transform infrared (ATR-FTIR) spectra of the pure Cannabis sativa ethanolic extract (CEE) and empty and CEE-loaded chitosan-coated nanocapsules (NCs) prepared with medium-chain triglyceride (MCT) oil or olive oil as the lipid core. For each sample, the raw spectrum was deconvoluted over the 4000–500 cm^{-1} region using linear baseline correction and Gaussian–Lorentzian peak fitting. In each figure, the original spectrum (red trace) is shown overlaid with the individually resolved component bands; the center position, height, full width at half height (FWHH), and, where available, the integrated area of each resolved band are listed in the accompanying table. These data support the band assignments summarized in Table 1 of the main manuscript.

Pure Cannabis sativa Ethanolic Extract (CEE)

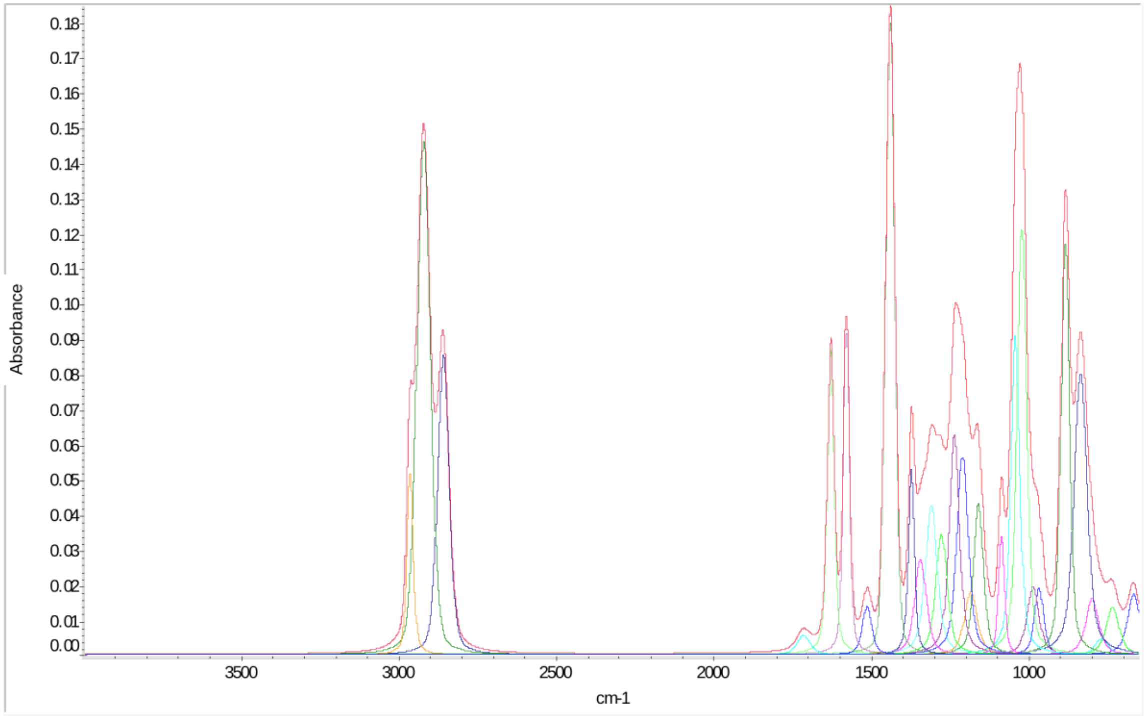

Figure S1. Deconvoluted ATR-FTIR spectrum of the pure Cannabis sativa ethanolic extract (CEE). The original spectrum (red trace) is shown overlaid with the individually resolved Gaussian–Lorentzian component bands obtained after deconvolution over the 4000–500 cm^{-1} region. The spectrum shows bands attributable to phenolic cannabinoids (C–O stretching and aromatic C=C vibrations), their aliphatic pentyl side chains (C–H stretching, 2800–3000 cm^{-1}), and co-extracted terpenoid constituents (out-of-plane =C–H bending, 800–900 cm^{-1}).

Table S1. Center position, height, and full width at half height (FWHH) of the individual bands resolved from the deconvoluted ATR-FTIR spectrum of the pure CEE (Figure S1).

Peak #	Center (cm^{-1})	Height (a.u.)	FWHH (cm^{-1})
33	3080.9	0.0000	43.3
32	2965.5	0.0514	23.4
31	2922.0	0.1462	50.0
30	2859.3	0.0855	43.0
29	2808.0	0.0000	42.4
28	1715.2	0.0055	49.2
27	1628.8	0.0867	25.0
26	1579.4	0.0919	22.6
25	1513.7	0.0136	34.3
24	1478.0	0.0012	48.2
23	1440.0	0.1798	34.0
22	1373.3	0.0529	22.6
21	1344.2	0.0272	43.7
20	1310.0	0.0424	45.0
19	1277.8	0.0344	42.7
18	1236.9	0.0627	38.0
17	1210.8	0.0562	42.7
16	1184.3	0.0185	47.1
15	1160.1	0.0431	35.9
14	1132.6	0.0000	45.3
13	1087.3	0.0338	21.0
12	1043.5	0.0907	33.0
11	1022.4	0.1214	35.0
10	986.3	0.0189	44.6
9	968.6	0.0190	33.5

Peak #	Center (cm^{-1})	Height (a.u.)	FWHH (cm^{-1})
8	916.2	0.0000	43.9
7	884.6	0.1171	31.5
6	836.1	0.0798	48.2
5	799.1	0.0160	48.1
4	769.9	0.0047	48.0
3	734.9	0.0136	48.2
2	705.8	0.0001	49.0
1	667.2	0.0172	47.8

Empty MCT Oil Nanocapsules (NCs)

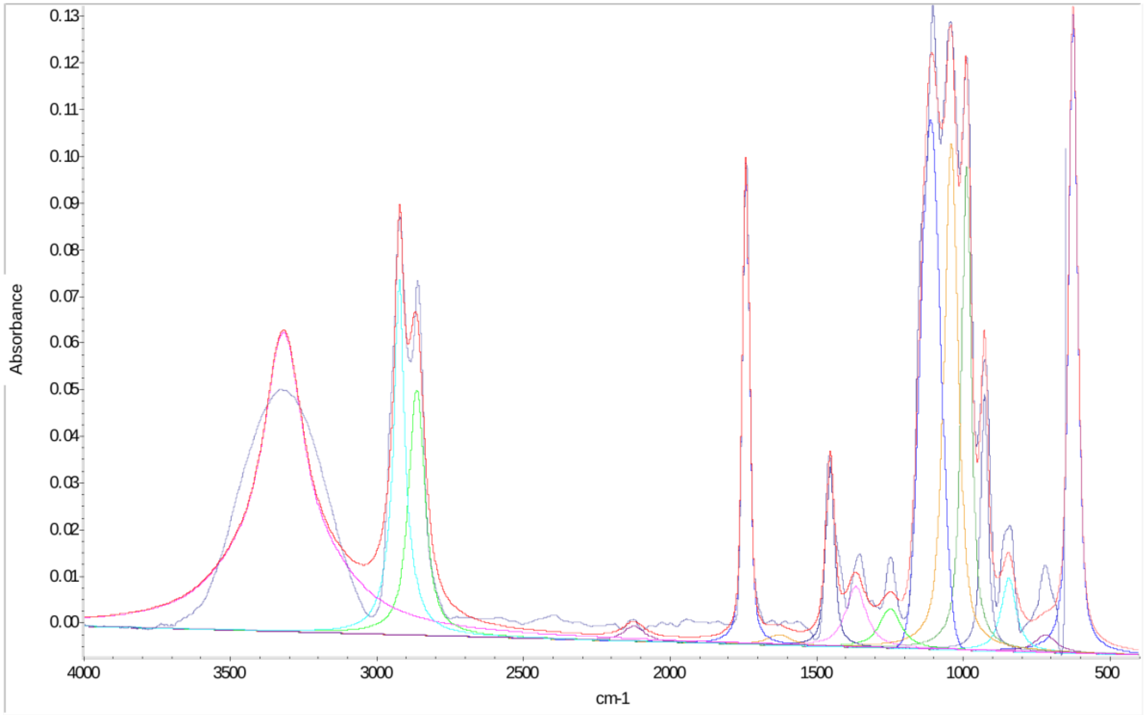

Figure S2. Deconvoluted ATR-FTIR spectrum of empty chitosan-coated MCT oil nanocapsules (NCs). The profile is dominated by the ester carbonyl stretching of the triglyceride core (~1740 cm⁻¹) and the aliphatic C–H stretching of the medium-chain fatty acid chains (~2924/2864 cm⁻¹), together with the broad O–H/N–H stretching band (~3320 cm⁻¹) arising from chitosan and Tween® 80.

Table S2. Center position, height, FWHH, and area of the individual bands resolved from the deconvoluted ATR-FTIR spectrum of the empty MCT oil NCs (Figure S2).

Peak #	Center (cm⁻¹)	Height (a.u.)	FWHH (cm⁻¹)	Area (a.u.)
21	3319.6	0.0643	198.1	22.40
20	2924.5	0.0763	43.5	5.79
19	2864.7	0.0526	66.4	5.05
18	2123.7	0.0033	85.7	0.41
17	1740.1	0.1033	28.5	3.85
16	1625.8	0.0023	97.4	0.33
15	1513.4	0.0000	99.9	0.00

Peak #	Center (cm⁻¹)	Height (a.u.)	FWHH (cm⁻¹)	Area (a.u.)
14	1453.2	0.0385	35.0	1.87
13	1365.5	0.0131	81.5	1.59
12	1295.7	0.0000	99.5	0.00
11	1247.9	0.0085	87.7	1.13
10	1144.5	0.0000	84.2	0.00
9	1110.5	0.1136	79.2	9.58
8	1040.1	0.1087	56.6	8.48
7	988.2	0.1039	38.9	5.92
6	927.1	0.0548	28.3	2.27
5	907.8	0.0000	101.3	0.00
4	844.0	0.0159	60.0	1.42
3	736.5	0.0000	89.8	0.00
2	718.0	0.0038	88.1	0.50
1	624.1	0.1370	37.3	6.84

CEE-Loaded MCT Oil Nanocapsules (NCs)

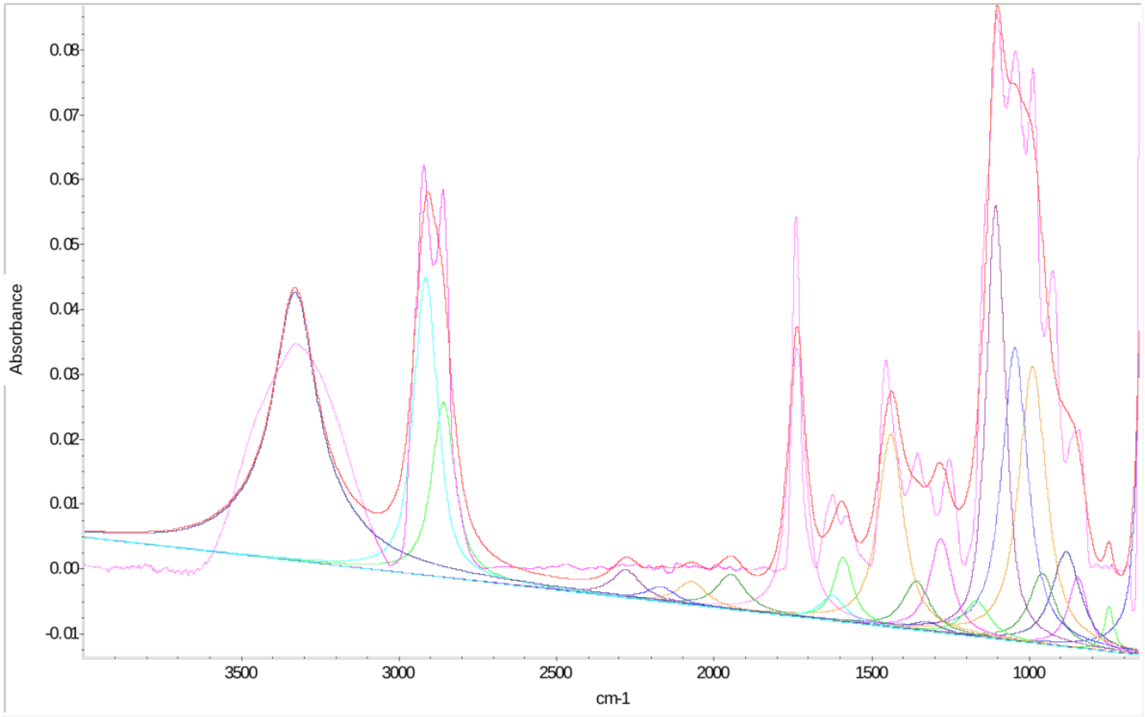

Figure S3. Deconvoluted ATR-FTIR spectrum of CEE-loaded chitosan-coated MCT oil nanocapsules. Relative to the empty NCs (Figure S2), the spectrum retains the core triglyceride and shell signatures while showing the shifts and attenuation of the extract's aromatic and O–H/N–H bands discussed in Section 3.4 of the main manuscript, consistent with successful encapsulation of the CEE within the lipid core.

Table S3. Center position, height, FWHH, and area of the individual bands resolved from the deconvoluted ATR-FTIR spectrum of the CEE-loaded MCT oil NCs (Figure S3).

Peak #	Center (cm^{-1})	Height (a.u.)	FWHH (cm^{-1})	Area (a.u.)
36	3833.7	0.0000	81.3	0.00
35	3780.8	0.0000	54.7	0.00
34	3713.9	0.0000	99.0	0.00
33	3666.9	0.0000	96.9	0.00
32	3626.0	0.0000	106.7	0.00
31	3597.6	0.0000	91.7	0.00
30	3330.0	0.0415	180.0	12.36
29	3055.1	0.0000	90.4	0.00
28	2915.8	0.0461	89.9	5.82

Peak #	Center (cm^{-1})	Height (a.u.)	FWHH (cm^{-1})	Area (a.u.)
27	2859.2	0.0271	87.6	3.54
26	2279.6	0.0043	111.0	0.72
25	2168.4	0.0023	110.7	0.39
24	2070.2	0.0036	111.5	0.62
23	1945.5	0.0054	111.1	0.91
22	1804.1	0.0000	111.5	0.00
21	1736.5	0.0414	65.2	4.02
20	1624.1	0.0039	94.5	0.57
19	1590.1	0.0100	72.3	1.10
18	1535.7	0.0000	89.4	0.00
17	1499.5	0.0000	65.8	0.00
16	1438.8	0.0298	96.2	4.26
15	1357.5	0.0076	99.7	1.15
14	1325.1	0.0015	109.0	0.25
13	1280.1	0.0145	85.2	1.86
12	1216.8	0.0000	94.1	0.00
11	1166.9	0.0055	102.7	0.85
10	1105.9	0.0670	71.8	6.73
9	1045.3	0.0453	97.4	6.57
8	989.1	0.0427	103.6	6.54
7	958.0	0.0109	102.1	1.68
6	882.3	0.0146	108.8	2.35
5	845.9	0.0110	72.6	1.21
4	786.0	0.0000	85.1	0.00
3	746.7	0.0069	37.6	0.40
2	710.0	0.0000	67.7	0.00
1	610.6	0.1302	63.1	10.70

Empty Olive Oil Nanocapsules (NCs)

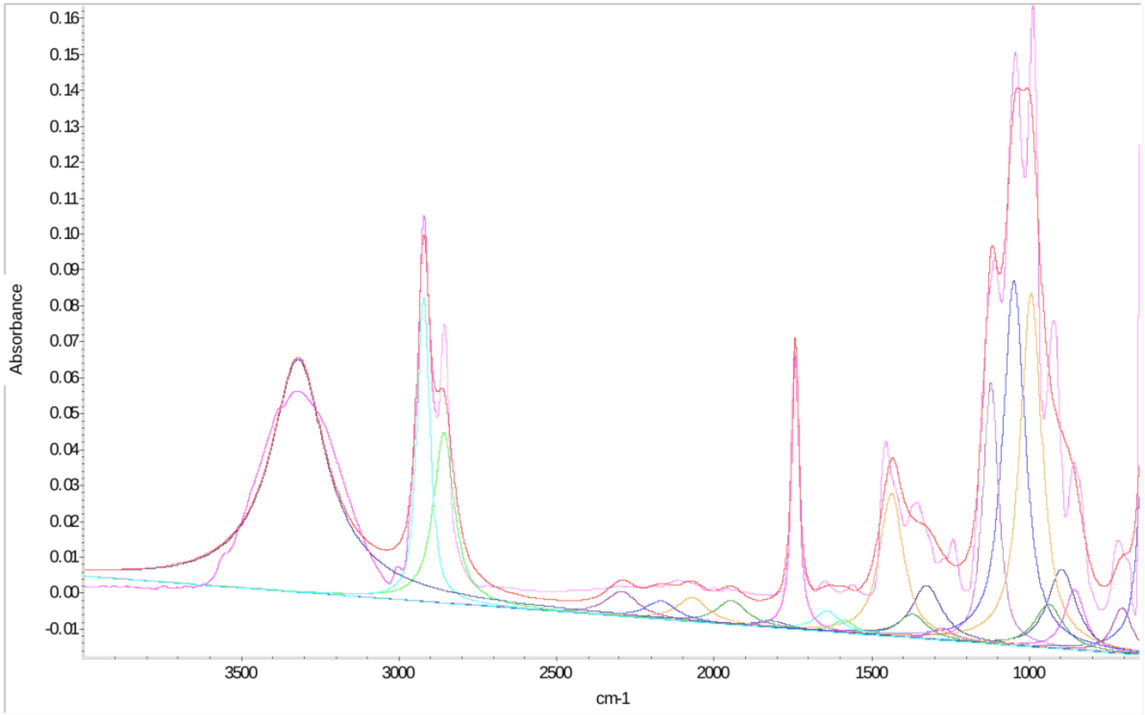

Figure S4. Deconvoluted ATR-FTIR spectrum of empty chitosan-coated olive oil nanocapsules. As for the MCT oil NCs, the spectrum is dominated by the ester carbonyl (~1740 cm⁻¹) and aliphatic C–H stretching bands of the triglyceride core, together with the O–H/N–H stretching band of the chitosan/Tween® 80 shell; the greater degree of unsaturation of olive oil is reflected in the relative band widths and intensities compared with the MCT oil system.

Table S4. Center position, height, FWHH, and area of the individual bands resolved from the deconvoluted ATR-FTIR spectrum of the empty olive oil NCs (Figure S4).

Peak #	Center (cm^{-1})	Height (a.u.)	FWHH (cm^{-1})	Area (a.u.)
36	3841.8	0.0000	84.1	0.00
35	3774.2	0.0000	71.2	0.00
34	3722.0	0.0000	104.2	0.00
33	3672.5	0.0000	103.6	0.00
32	3626.8	0.0000	109.8	0.00
31	3571.6	0.0000	97.2	0.00
30	3320.1	0.0648	215.0	22.34

Peak #	Center (cm⁻¹)	Height (a.u.)	FWHH (cm⁻¹)	Area (a.u.)
29	3076.0	0.0000	94.3	0.00
28	2922.5	0.0846	49.3	5.90
27	2858.0	0.0476	78.2	5.65
26	2293.7	0.0070	121.6	1.32
25	2168.7	0.0051	117.0	0.92
24	2068.8	0.0067	117.8	1.21
23	1943.7	0.0067	117.3	1.19
22	1817.0	0.0019	116.7	0.34
21	1741.8	0.0763	29.6	3.35
20	1640.0	0.0057	99.6	0.88
19	1587.4	0.0036	80.8	0.45
18	1539.5	0.0000	97.3	0.00
17	1489.8	0.0000	78.4	0.00
16	1436.5	0.0400	91.1	5.42
15	1369.9	0.0068	105.7	1.09
14	1324.8	0.0150	113.3	2.57
13	1275.3	0.0033	92.8	0.47
12	1220.9	0.0012	100.6	0.18
11	1162.9	0.0000	107.3	0.00
10	1121.7	0.0729	58.9	6.06
9	1048.2	0.1019	83.7	12.73
8	993.7	0.0986	82.7	12.21
7	937.5	0.0123	108.8	2.03
6	896.3	0.0222	108.8	3.58
5	855.7	0.0170	76.0	1.96
4	797.7	0.0000	89.8	0.00
3	752.4	0.0000	59.9	0.00
2	705.2	0.0127	75.4	1.47
1	604.4	0.1436	66.4	12.84

CEE-Loaded Olive Oil Nanocapsules (NCs)

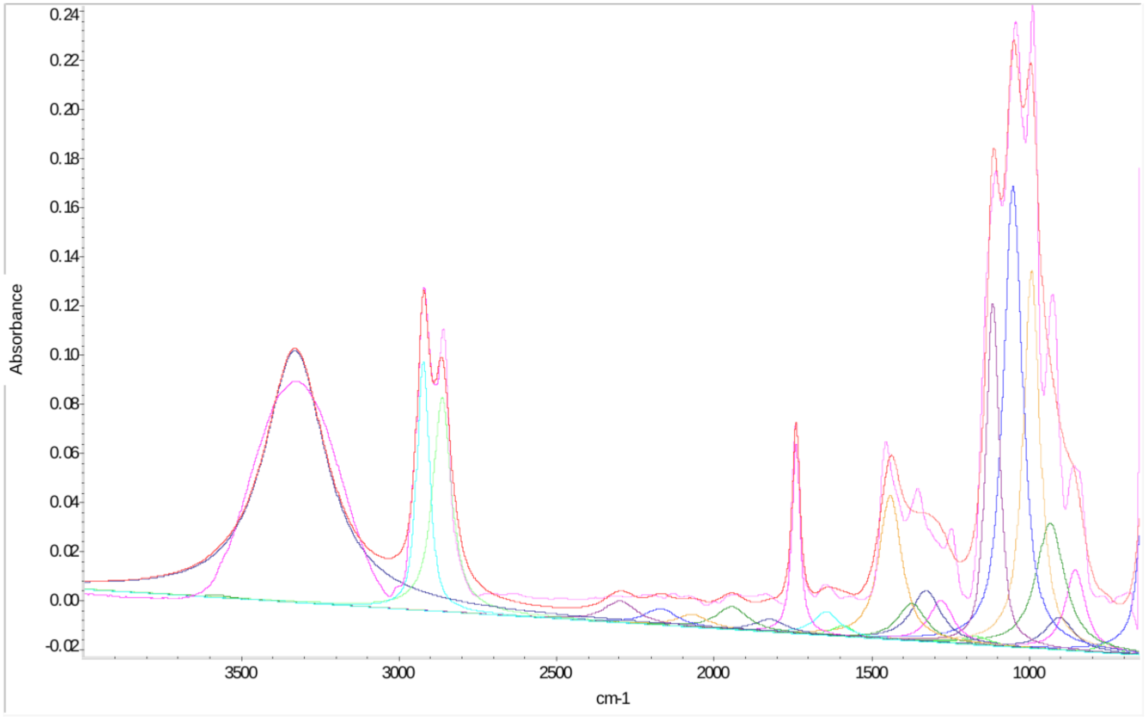

Figure S5. Deconvoluted ATR-FTIR spectrum of CEE-loaded chitosan-coated olive oil nanocapsules. As discussed in Section 3.4 of the main manuscript, this formulation shows more pronounced O–H/N–H (~3330 cm⁻¹) and aromatic C=C (~1600 cm⁻¹) bands relative to the MCT oil system, consistent with a greater interfacial localization of CBD within the more fluid, unsaturated olive oil core.

Table S5. Center position, height, FWHH, and area of the individual bands resolved from the deconvoluted ATR-FTIR spectrum of the CEE-loaded olive oil NCs (Figure S5).

Peak #	Center (cm^{-1})	Height (a.u.)	FWHH (cm^{-1})	Area (a.u.)
36	3841.3	0.0000	83.1	0.00
35	3774.0	0.0000	75.0	0.00
34	3723.6	0.0000	104.9	0.00
33	3673.7	0.0000	105.0	0.00
32	3629.3	0.0000	108.0	0.00
31	3567.6	0.0010	94.9	0.14
30	3330.4	0.1027	236.4	37.50

Peak #	Center (cm⁻¹)	Height (a.u.)	FWHH (cm⁻¹)	Area (a.u.)
29	3086.9	0.0000	94.4	0.00
28	2923.4	0.1014	47.8	6.85
27	2862.7	0.0872	69.6	9.23
26	2298.4	0.0089	125.4	1.73
25	2169.1	0.0069	118.8	1.26
24	2068.6	0.0053	119.4	0.96
23	1942.3	0.0094	119.2	1.71
22	1820.6	0.0053	118.2	0.94
21	1739.4	0.0773	31.1	3.58
20	1642.3	0.0096	100.9	1.50
19	1590.3	0.0045	82.9	0.56
18	1539.4	0.0000	98.8	0.00
17	1490.6	0.0000	81.3	0.00
16	1440.3	0.0588	85.6	7.48
15	1371.9	0.0153	107.0	2.49
14	1326.6	0.0208	113.6	3.57
13	1279.4	0.0172	92.3	2.41
12	1220.7	0.0000	102.1	0.00
11	1166.1	0.0034	107.9	0.56
10	1115.4	0.1399	51.6	9.95
9	1051.2	0.1879	72.2	20.06
8	992.1	0.1540	62.1	14.56
7	933.1	0.0518	108.4	8.50
6	904.0	0.0133	109.2	2.16
5	853.7	0.0333	75.8	3.84
4	802.0	0.0000	92.3	0.00
3	752.7	0.0042	64.4	0.42
2	701.8	0.0001	78.8	0.01
1	602.8	0.1611	67.7	14.82

Table S6. Acquisition parameters and quantitative analysis of all confocal images.

Nikon C1 confocal system, Plan Apo VC 60×/1.40 NA oil objective, 1024 × 1024 px, 12-bit; 488 nm laser at 80.8% (405 and 543 nm at minimum power); pinhole 153.8 µm. Channels: 515/30 nm (green) and 605/75 nm (red). P99: 99th percentile of the background-subtracted image (arbitrary units). [Confirm sample identities.]

Image file	Sample	Date	Field (µm)	Pixel (µm)	Dwell (µs)	Gain 515/30	Gain 605/75	P99 515/30	P99 605/75	Used in
nano_coco_cargadas	CBD-loaded MCT oil NCs	2025-10-07	80	0.078	6.00	6.80	6.80	2941	1849	Figure 11A
nano_coco_vacias	Empty MCT oil NCs	2025-10-07	89	0.087	6.00	6.80	6.80	874	306	Figure 11B
oliva	CBD-loaded olive oil NCs	2025-10-07	73	0.071	6.00	6.80	6.80	1733	1275	Figure 11C
oliva001	CBD-loaded olive oil NCs (field 2)	2025-10-07	107	0.104	6.00	6.50	6.80	1743	1554	Figure S6A
oliva_vacio1	Empty olive oil NCs	2025-10-07	212	0.207	6.00	6.80	6.80	660	465	Figure 11D
oliva_vacio2	Empty olive oil NCs (field 2)	2025-10-07	100	0.098	6.00	6.80	6.80	72	96	Figure S6B
oliva_vacio3	Empty olive oil NCs (field 3)	2025-10-07	212	0.207	6.00	6.80	6.80	485	321	Figure S6C
colorante	Fast Blue alone	2025-10-07	88	0.086	6.00	6.80	6.80	115	36	Figure S6D
quitosan	Chitosan alone	2025-11-07	212	0.207	6.00	6.80	6.50	6	4	Figure S6E
oliva_bco	Olive oil blank	2025-11-07	212	0.207	6.00	6.80	6.50	12	12	Figure S6F
oliva_bco1	Olive oil blank (field 2)	2025-11-07	18	0.017	6.00	6.80	6.50	9	8	Table S6 only
Coco	MCT oil blank	2025-11-07	212	0.207	6.00	6.80	6.50	10	10	Figure S6G

Notes: (1) The blanks acquired on 2025-11-07 used a red gain of 6.5 instead of 6.8, and oliva001 a green gain of 6.5 instead of 6.8; the blanks lie at the background level, so the conclusions are not affected. (2) Ratios of loaded to empty NCs (October session): 515/30 nm, 3.4× for MCT oil and 4.3× for olive oil; 605/75 nm, 6.0× for MCT oil and 4.8× for olive oil.

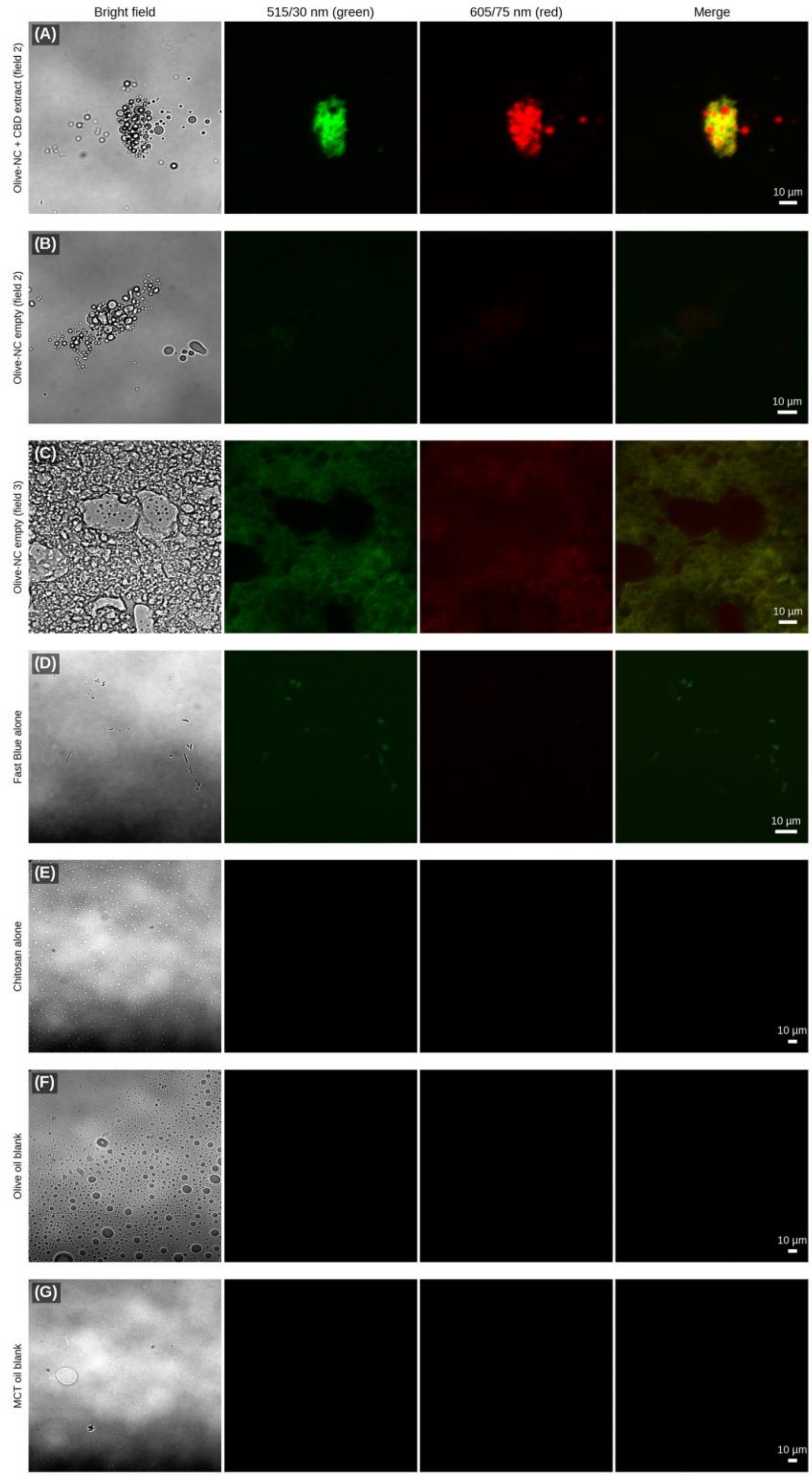

Figure S6. Confocal laser scanning microscopy of additional fields and of controls. (A) CBD-loaded olive oil NCs (field 2); (B, C) empty olive oil NCs (fields 2 and 3; C is a 512 × 512-pixel crop); (D) Fast Blue alone; (E) chitosan alone; (F) olive oil blank; (G) MCT oil blank. Columns: bright field, 515/30 nm (green), 605/75 nm (red), merge. Acquisition and display settings as in Figure 11 (20–2200 ADU for 515/30 nm, 20–1600 ADU for 605/75 nm); panels E–G were acquired in a separate session (Table S6). Scale bars: 10 µm.

Figure S7. Concentration-dependent effects of CEE-loaded NCs, empty NCs and CEE oil on Vero cell viability and CHIKV-induced cytopathic effect. (A,D) CEE-loaded MCT oil NCs and CEE-loaded olive oil NCs; (B,E) empty MCT oil NCs and empty olive oil NCs; (C,F) CEE dissolved in MCT oil (CEE MCT oil) and in olive

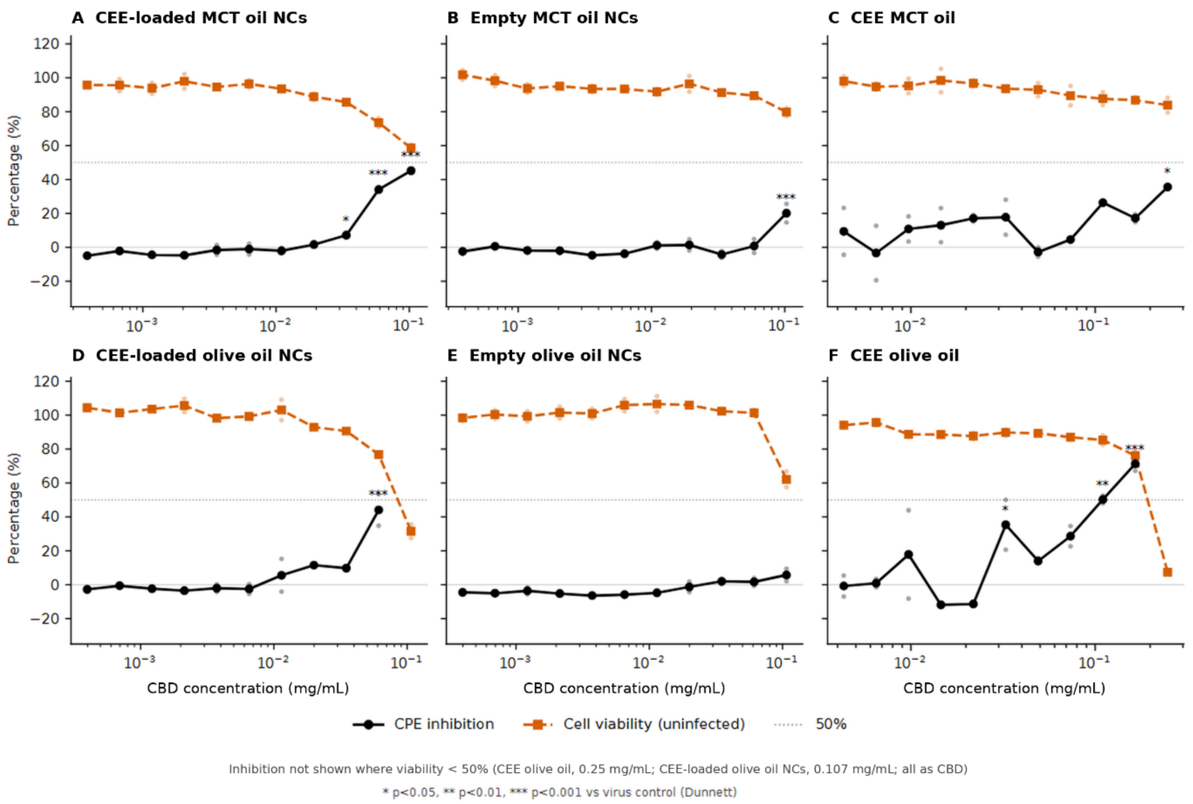

oil (CEE olive oil). Orange dashed lines with squares show the viability of uninfected Vero cells (% of the cell control); black solid lines with circles show the inhibition of CHIKV-induced CPE in infected cells (%), calculated as described in Section 2.15.3. Lines join the mean values and light points are individual replicates. Concentrations are plotted on a logarithmic scale and expressed in mg/mL of CBD; for CEE dissolved in oil, the CBD concentration was calculated from the CBD content of the extract (48%). The dotted line marks 50%. CPE inhibition is not shown where viability was below 50% (CEE olive oil, 0.12 mg/mL; CEE-loaded olive oil NCs, 0.107 mg/mL). * $p < 0.05$, ** $p < 0.01$ and *** $p < 0.001$ versus the virus control (Dunnett's test).
